# Pharmacogenetic, Clinical, Prescribing, and Demographic Predictors of Sertraline Pharmacokinetics

**DOI:** 10.3390/pharmaceutics18091133

**Published:** 2026-09-09

**Authors:** Carla González de la Cruz, Levin Thomas, Carmen Mata-Martín, Juan Antonio Villatoro-García, Fernando de Andrés, Idian González-Rodríguez, Idilio González-Martínez, Eva M. Peñas-Lledó, Adrián LLerena

**Affiliations:** 1 Personalised Medicine and Mental Health Unit, University Institute for Biosanitary Research of Extremadura (INUBE), 06080 Badajoz, Spain; carla.gonzalezd@unex.es (C.G.d.l.C.); levintx@unex.es (L.T.); mariadelcarmen.mata@salud-juntaex.es (C.M.-M.); juan.villatorogarcia@externos.salud-juntaex.es (J.A.V.-G.); idian.gonzalez@salud-juntaex.es (I.G.-R.); idilio.gonzalez@salud-juntaex.es (I.G.-M.); 2 Clinical Pharmacology Service, Pharmacogenomics and Personalized Medicine Unit, Badajoz University Hospital, Extremadura Health Service (SES), 06006 Badajoz, Spain; 3 Department of Analytical Chemistry and Food Technology, Faculty of Pharmacy, University of Castilla-La Mancha, 02008 Albacete, Spain; fernando.deandres@uclm.es; 4 Regional Institute of Applied Scientific Research IRICA, University of Castilla-La Mancha, 13004 Ciudad Real, Spain; 5 Psychiatry Unit, San Pedro de Alcántara Hospital, Extremadura Health Service (SES), 10002 Cáceres, Spain; 6 Psychiatry Unit, Llerena Hospital, Extremadura Health Service (SES), 06900 Llerena, Spain

**Keywords:** *CYP2C19*, pharmacokinetics, pharmacogenetics, sertraline

## Abstract

**Background**: Interindividual variability in sertraline pharmacokinetics remains substantial, but the relative contributions of pharmacogenetic (PGx), demographic, clinical, and prescribing determinants have not been comprehensively evaluated in routine clinical practice. The study aimed to evaluate PGx, demographic, clinical, and prescribing determinants of sertraline plasma concentrations, sertraline plasma concentration-to-dose ratio (C/D), and non-dose-normalized sertraline-to-norsertraline metabolic ratio (MR), with log_10_-transformed dose-normalized sertraline/norsertraline metabolic ratio (LogMR_DN_) and log_10_-transformed MR (LogMR) evaluated as exploratory derived pharmacokinetic outcomes in real-world clinical settings. **Methods**: This prospective real-world PGx study included 153 patients receiving sertraline therapy who participated in the MedeA clinical PGx implementation program. Associations of genotype-predicted phenotypes for *CYP2C19*, *CYP2B6*, *CYP2D6*, *CYP2C9*, and *CYP3A4*, *CYP2C:TG* haplotype status, demographic, clinical, and prescribing characteristics with sertraline concentration, C/D, MR, and LogMR_DN_ were evaluated using univariable analyses. Separate multivariable linear regression models were fitted for these outcomes and LogMR using 140 complete cases. **Results**: *CYP2C19* gPM status was associated with higher sertraline concentrations (β = 72.10, 95% CI: 21.36–122.84; *p* = 0.01). Although a higher sertraline C/D ratio was observed in the conventional multivariable model with *CYP2C19* gPM (β = 1.46, 95% CI: 0.85–2.08; *p* < 0.001), this association was not retained after HC3 heteroscedasticity-robust standard errors were applied (β = 1.46, 95% CI: −0.09–3.02; *p* = 0.07). In a small exploratory subgroup analysis, patients with combined *CYP2C19* and *CYP2B6* poor-metabolizer status (n = 2) had sertraline concentrations exceeding 150 ng/mL. **Conclusions**: The study findings provide further insights into the relevance of *CYP2C19* in sertraline pharmacokinetics.

## 1. Introduction

Sertraline is a widely prescribed selective serotonin reuptake inhibitor (SSRI) used for the treatment of several mental health disorders including major depressive disorder, panic disorder, obsessive–compulsive disorder, post-traumatic stress disorder, premenstrual dysphoric disorder, and social anxiety disorder. Its broad clinical use reflects a favorable balance of efficacy, tolerability, and a generally lower propensity for clinically important drug–drug interactions (DDIs) than several other antidepressants [1,2]. Although a definitive concentration–response range has not been established, therapeutic drug monitoring (TDM) guidance initially proposed a sertraline reference range of 10–150 ng/mL and a laboratory alert concentration of 300 ng/mL [3], while a recent report suggests that sertraline concentrations < 25 ng/mL may be associated with a lower clinical response [4]. However, recent Arbeitsgemeinschaft für Neuropsychopharmakologie und Pharmakopsychiatrie (AGNP) TDM task force guidelines have specified a sertraline therapeutic reference range of 10–50 ng/mL for depression, with a laboratory alert level of 150 ng/mL [5]. Although sertraline exhibits linear pharmacokinetics (PK) across the therapeutic dose range, considerable interindividual variability in plasma concentrations remains, reflecting variability in the functional activity of metabolic enzymes, pharmacogenetics (PGx) factors, concomitant medications, and other clinical characteristics [6,7,8]. This variability may be associated with adverse drug reactions (ADRs) due to higher serum concentrations [9]. In this way, identifying the determinants of sertraline exposure is therefore clinically relevant for optimizing dose selection, minimizing avoidable ADRs, and improving treatment effectiveness.

Sertraline undergoes extensive metabolism via hepatic *N*-demethylation, mediated by overlapping contributions from *CYP450* enzymes including *CYP2C19*, *CYP2B6*, *CYP2D6*, *CYP2C9*, and *CYP3A4*, to form its major active metabolite, norsertraline (desmethylsertraline), which generally attains higher steady-state plasma concentrations than the parent drug [10]. Genetic variation within these enzymes contributes to variability in sertraline PK, with the strongest evidence supporting *CYP2C19* and *CYP2B6* as clinically actionable pharmacogenes [11,12], while the contribution of *CYP2D6*, *CYP2C9*, and *CYP3A4*, remains uncertain. More recently, the *CYP2C* haplotype defined by the co-occurrence of rs2860840T and rs11188059G (*CYP2C:TG*) has been associated with an increased rate of *CYP2C19*-mediated sertraline metabolism and, consequently, lower sertraline exposure, suggesting the possibility that haplotype-informed PGx analyses could further refine prediction of sertraline metabolism beyond conventional *CYP2C19* metabolizer phenotype classification [13]. Most previous studies have focused on individual pharmacogenes, predominantly *CYP2C19*. Although PGx recommendations are available, showing the combined utility of *CYP2C19* and *CYP2B6* for sertraline dosing [14], the relative contribution of multiple pharmacogenes alongside demographic, clinical, and prescribing factors has not been comprehensively evaluated within the same real-world patient cohort. Because sertraline metabolism involves multiple CYP pathways and may also be influenced by clinical and prescribing factors, evaluating these determinants within a single multivariable model may help better define their relative contribution to PK variability. Measured sertraline plasma concentration provides a clinically interpretable index of systemic drug exposure against which the contributions of PGx and non-genetic determinants can be evaluated. Sertraline concentration-to-dose ratio (C/D) accounts for differences in prescribed sertraline daily dose and provides an index of dose-adjusted sertraline exposure. The non-dose-normalized sertraline-to-norsertraline metabolic ratio (MR) reflects the combined disposition of the parent drug (sertraline) and its major metabolite (norsertraline). Log_10_-transformed dose-normalized MR (LogMR_DN_) and log_10_-transformed MR (LogMR) were additionally evaluated as exploratory derived PK outcomes. Together, these PK outcomes enable a complementary evaluation of the determinants of sertraline exposure and overall metabolic disposition. The integration of PGx information with plasma concentration data may further improve understanding of variability in sertraline concentrations and support individualized treatment strategies [15]. The aim of this study was to evaluate the independent and relative contributions of *CYP2C19*, *CYP2B6*, *CYP2D6*, *CYP2C9*, *CYP3A4*, the *CYP2C:TG* haplotype, and relevant demographic, clinical, and prescribing characteristics to sertraline plasma concentration, C/D, and MR, with LogMR_DN_ and LogMR examined as exploratory derived PK outcomes, in patients enrolled in a real-world clinical PGx implementation program.

## 2. Materials and Methods

### 2.1. Study Design, Patient and Ethical Committee Approval

The study included 153 Spanish patients enrolled in the MedeA program, a real-world clinical PGx implementation initiative that integrates pre-emptive PGx testing into routine clinical care [16], between February 2022 and April 2026, following the same clinical workflow previously described [17]. Patients were included in the study if they were residents of Extremadura, attended the Extremadura Health Service (SES), were prescribed sertraline, self-reported adherence to the prescribed treatment regimen during the clinical interview, and had documentation of the prescribed sertraline dose and concomitant medications. Pregnancy was an exclusion criterion for the current study. Patients who had received sertraline for less than seven days were also excluded. Written informed consent was obtained from all patients prior to their inclusion in the study. Clinical and demographic data (including age, sex, body mass index [BMI], smoking status, comorbidities, concomitant medications, laboratory data, and polypharmacy (≥5 medications)) were collected during clinical assessment and extracted from electronic medical records. The number of diagnoses and concomitant medications was assessed from electronic health-record entries at the time of blood sampling. At the time of sertraline prescribing and dose selection, the analyzed PGx results were not yet available to prescribers; therefore, treatment and dosing decisions were not informed by these results. The principles of the Declaration of Helsinki were adhered to, and the study received approval from the Ethics Committee for investigation with Medicines of Cáceres (CEIm) [No. 052-2021].

### 2.2. Genotyping

Genomic DNA was isolated from peripheral blood samples and genotyped for *CYP2C19*, *CYP2B6*, *CYP2D6*, *CYP2C9*, and *CYP3A4* using commercially available TaqMan^®^ allelic discrimination assays (Applied Biosystems, Foster City, CA, USA). The single-nucleotide variants included in each pharmacogene that define the alleles of interest are described in Supplementary Table S1 (Supplementary File S1). Polymerase chain reaction (PCR) amplification and allele discrimination were performed according to the manufacturer’s recommendations using previously described methodology [18]. All assays included negative (no DNA) and positive (heterozygous and/or homozygous) control samples to ensure assay accuracy. Copy number variation analysis (*CYP2D6* gene duplications and CYP2D6*5 deletions) was performed using a previously described methodology [19]. Genotype-predicted metabolizer phenotypes for the analyzed genes were classified according to the Clinical Pharmacogenetics Implementation Consortium (CPIC) guidelines [14,20]. For *CYP2D6*, *CYP2C9*, and *CYP2C19*, the genotype-to-phenotype assignment was performed as previously described [21]. In the case of *CYP2B6*, individuals carrying two increased-function alleles were classified as genotype-predicted ultrarapid metabolizers (gUMs) (*4/*4), whereas carriers of one normal-function allele and one increased-function allele were classified as genotype-predicted rapid metabolizers (gRMs) (*1/*4). Individuals carrying two normal-function alleles were classified as genotype-predicted normal metabolizers (gNMs) (*1/*1). Individuals carrying one normal-function allele and one decreased-function or no-function allele, or one increased-function allele and one decreased-function or no-function allele, were classified as genotype-predicted intermediate metabolizers (gIMs) (*1/*6, *4/*9). Individuals carrying two decreased-function alleles, two no-function alleles, or one decreased-function and one no-function allele were classified as genotype-predicted poor metabolizers (gPMs) (*6/*6) [14]. Genotype-predicted metabolizer phenotype assessment for *CYP3A4* was performed in accordance with the Dutch Pharmacogenetics Working Group (DPWG) clinical guidelines [22]. *CYP2C* haplotypes were defined using the allele order *rs2860840*/*rs11188059*; the ‘TG’ haplotype corresponds to *rs2860840* = T and *rs11188059* = G [13].

### 2.3. PK Profiling

Blood samples were collected immediately before the next scheduled sertraline dose administration to determine pre-dose sertraline and norsertraline plasma concentrations, and plasma was separated by centrifugation (3500 rpm, 13 min) and stored at −20 °C until analysis. Quantification of sertraline and norsertraline was performed using a validated commercial assay kit supplied by Chromsystems (Chromsystems Instruments & Chemicals GmbH, Munich, Germany) [23]. Plasma samples obtained from patients, as well as calibration and quality control samples, were prepared according to the manufacturer’s protocol. Briefly, 50 μL of plasma was placed into a 1.5 mL vial, mixed with 25 μL of extraction buffer (Ref. 92005), and incubated for 2 min. Next, 250 μL of internal standard solution was added, and the mixture was vortexed for 30 s. Samples were then centrifuged immediately at 13,000 rpm for 7 min. After centrifugation, 150 μL of the supernatant was transferred to an amber glass vial and combined with 150 μL of dilution buffer 1 (Ref. 92007).

For the calibration curve, 600 µL of internal standard (IS) at a concentration of 10 ppm (10 µL aripiprazole-d8 (1 mg/mL) + 990 µL MeOH) was used. It was prepared to a final volume of 10 mL with a precipitating agent (Ref. 92003). The calibration standards and QC samples were prepared by adding the respective working solutions to blank plasma, yielding 10 different concentrations for both sertraline and norsertraline: 0.30, 0.60, 1.20, 2.40, 4.81, 9.62, 19.23, 38.46, 76.92, and 153.85 ng/mL. The final concentration of the IS was 230.77 ng/mL. Samples exceeding the upper limit of the calibration curve were diluted with blank/analyte-free plasma and re-analyzed within the validated calibration range. All calibration and quality control samples were prepared immediately prior to analysis.

Precision and accuracy were evaluated in six replicates at three concentration levels (low, medium, and high) in a single analytical run within a single day. Coefficient of variation (CV, %) was evaluated for estimated precision, and accuracy was calculated as a percentage of the nominal concentration (%). The CV should not exceed 15% except for the limit of quantification (LoQ), where it should not exceed 20% of the CV (Table 1).

Calibration curves for sertraline and norsertraline were plotted as peak area vs. concentration for each analyte. This was tested in triplicate, and linearity was assessed using linear regression and the correlation coefficient (R^2^). The lower limit of quantification (LLOQ) for sertraline was 6.4364 ng/mL, and for norsertraline, 6.42 ng/mL.

Chromatographic analysis was carried out using an Agilent 1200 Series HPLC system (Agilent Technologies, Santa Clara, CA, USA), equipped with a binary pump, autosampler, degasser, and column compartment. Separation was achieved on a Chromsystems MassTox^®^ TDM MasterColumn A (50 mm × 2 mm i.d., 3 μm particle size; Ref. 92110) (Chromosystems Instruments & Chemicals GmbH, Gräfelfing, Germany), maintained at 30 °C throughout the analysis. Detection was performed with an API2000 triple quadrupole mass spectrometer (AB Sciex, Framingham, MA, USA) fitted with an atmospheric-pressure electrospray ionization (ESI) source and operated using Analyst software version 1.5.1.

### 2.4. Data Analysis

Continuous variables were assessed for normality using the Shapiro–Wilk test and are presented as median (Q1, Q3), whereas categorical variables are summarized as frequencies and percentages. Associations between prescribed daily sertraline dose and plasma concentrations of sertraline and norsertraline were evaluated using Spearman’s rank correlation. The MR was calculated individually for each patient as the sertraline plasma concentration divided by the corresponding norsertraline plasma concentration:


$$MR=\frac{Sertraline\;plasma\;concentration}{Norsertraline\;plasma\;concentration}$$

MR was subsequently log_10_-transformed to obtain LogMR.

The metabolic ratio dose-normalized MR_DN_ was calculated by dividing MR by the prescribed daily sertraline dose:

$$MRDN=\frac{MR}{Daily\;sertraline\;dose}$$

MR_DN_ was subsequently log_10_-transformed to obtain LogMR_DN_. Sertraline plasma concentration, sertraline C/D, and MR were evaluated as the main PK outcomes. LogMR_DN_ and LogMR were evaluated as exploratory derived PK outcomes. Univariable analyses were conducted for sertraline concentration, C/D, MR, and LogMR_DN_. LogMR was not included in the univariable group comparisons and was evaluated only in the exploratory multivariable analysis.

Univariable comparisons of sertraline concentration, C/D, MR, and LogMR_DN_ across genotype-predicted metabolizer groups, demographic, clinical, prescribing, and other subgroup variables were performed using one-way ANOVA or the Kruskal–Wallis test for comparisons involving more than two groups, and Welch’s t-test or the Mann–Whitney test for two-group comparisons, as appropriate based on the data distribution. Pairwise comparisons were performed using Tukey’s and Dunn’s multiple-comparisons tests following one-way ANOVA and Kruskal–Wallis analyses, respectively. Patient sertraline and norsertraline concentrations found below the LLOQ (n = 2 for sertraline and 7 for norsertraline) were imputed as one-half the LLOQ (LLOQ/2), a widely used approach for handling below-quantification-limit data in PK analyses [24,25], and were included as such in the descriptive, univariate, and multivariate analyses. Multivariable linear regression (*lm* function) analyses were performed to identify independent PGx, demographic, clinical, and prescribing determinants of the three main PK outcomes—sertraline concentration, C/D, and MR- and the two exploratory derived PK outcomes, LogMR_DN_ and LogMR. Covariates included genotype-predicted metabolizer phenotypes for *CYP2D6*, *CYP2C19*, *CYP2B6*, *CYP2C9*, and *CYP3A4*; *CYP2C:TG* haplotype status; age; sex; smoking status; and number of drugs. The number of diagnoses was not included concurrently due to potential collinearity with medication count. Multicollinearity was assessed using generalized variance inflation factors calculated with the car package in R. Multivariable model assumptions were assessed by visual inspection of residual vs. fitted plots and normal Q–Q plots of standardized residuals. Heteroscedasticity was evaluated using the studentized Breusch–Pagan (BP) test. To assess the robustness of the findings when heteroscedasticity was detected for a model, a sensitivity analysis was performed using HC3 heteroscedasticity-robust standard errors [26]. The statistical significance of the predictors was re-evaluated under this approach. All models included 140 complete cases. Concomitant CYP inhibitor medication exposure was reviewed and classified by inhibitor strength according to Flockhart’s Drug Interactions Table [27]. Analyses stratified by *CYP2C19* phenotype and *CYP2C:TG* haplotype status were descriptive because of the small sizes of the resulting subgroups and included sertraline concentration, C/D, MR, and LogMR_DN_. The combined *CYP2C19*/*CYP2B6* poor-metabolizer analysis was also descriptive and evaluated sertraline concentration and C/D without formal statistical testing. Sertraline concentration, C/D, MR, and LogMR_DN_ were summarized descriptively according to concomitant *CYP2D6* and *CYP2C19* inhibitor strength. Formal between-group comparisons were not performed because inhibitor categories overlapped and very few patients received strong inhibitors. CYP inhibitor variables were therefore not included in the multivariable models.

A *p* < 0.05 was considered to be statistically significant. All statistical analyses in the study were performed using GraphPad Prism (version 10.6.1) and R (version 4.5.1). Given that no primary hypothesis was prespecified and multiple PGx and clinical variables were evaluated, all analyses were considered exploratory and hypothesis-generating.

## 3. Results

### 3.1. Clinical and PK Profile of the Study Cohort

The study cohort consisted predominantly of older adults, with a median (Q1, Q3) age of 73 (61, 80.5) years, a BMI of 27.7 (24.5, 30.8) kg/m^2^, and a median of 10 (7, 13) medications. A total of 73.9% were women. The median prescribed sertraline dose was 50 mg/day (range: 25–300 mg/day). At the time of plasma sampling, the median (Q1, Q3) duration of sertraline treatment was 270 (105.5, 663) days. Plasma concentrations of sertraline and norsertraline were available for all 153 patients. The median (Q1, Q3) plasma sertraline concentration was 53.7 (29.9, 89.7) ng/mL, sertraline C/D was 0.8 (0.4, 1.2), plasma norsertraline concentration was 63.4 (32.8, 98.2) ng/mL, MR was 0.9 (0.7, 1.2), and LogMR_DN_ was −1.9 (−2.1, −1.7). Prescribed sertraline doses showed moderate positive correlations with both plasma sertraline (Spearman’s r = 0.41, 95% CI: 0.27 to 0.54; *p* < 0.0001) and norsertraline concentrations (Spearman’s r = 0.41, 95% CI: 0.26 to 0.54; *p* < 0.0001). A total of 18 (11.8%) had plasma sertraline concentrations above 150 ng/mL, whereas five (3.3%) had concentrations below 10 ng/mL. Genotype-predicted metabolizer group assignment was available for 153 patients for *CYP2C19* and *CYP2C9*, 152 for *CYP2B6* and *CYP3A4*, and 143 for *CYP2D6.* The *CYP2C:TG* haplotype status was available for 151 patients. Strong CYP inhibitor exposure occurred in only two patients for *CYP2D6* and one patient for *CYP2C19*. Moderate inhibitor exposure was also limited for *CYP2D6* (n = 1) but was more frequent for *CYP2C19* (n = 10), while weak inhibitor use was observed among 8 patients for *CYP2D6* and 65 patients for *CYP2C19*. No patient had any exposure to *CYP2C9* or *CYP2B6* inhibitors. Median (Q1, Q3) sertraline concentrations, sertraline C/D, MR and LogMR_DN_, by *CYP2D6* and *CYP2C19* inhibitor strength are presented in Supplementary Table S2.

### 3.2. Univariable Associations of Demographic, Clinical, and Prescribing Characteristics with Sertraline PK Outcomes

Univariable associations between demographic, clinical, and prescribing characteristics with the sertraline concentration, sertraline C/D, and MR are presented in Table 2, while corresponding associations with LogMR_DN_ are presented in Supplementary Table S3. Patients aged ≥ 65 years had a higher median sertraline C/D than those aged < 65 years (*p* = 0.02), but a lower median MR (*p* = 0.0004). Polypharmacy was associated with higher sertraline concentrations (*p* = 0.03) and sertraline C/D (*p* = 0.04), and lower MR (*p* = 0.01) and LogMR_DN_ (*p* = 0.04). Patients with ≥5 diagnoses also had lower MR (*p* = 0.0001) and lower LogMR_DN_ (*p* = 0.01) than those with <5 diagnoses. No statistically significant differences in any PK outcome were observed by sex, BMI, smoking status, ALT, AST, GGT, ALP, or urea concentration.

### 3.3. Distribution of PGx Alleles, Genotypes, and Metabolizer Groups

Allele and genotype distributions for *CYP2D6*, *CYP2C19*, *CYP2B6*, *CYP2C9*, and *CYP3A4* are presented in Supplementary Tables S4 and S5 (Supplementary File S1), respectively. The enrichment of *CYP2C19* and *CYP2B6* PMs among patients with sertraline concentrations > 150 ng/mL is interesting; however, given the relatively small size of the high-concentration subgroup, the findings should be interpreted with caution.

### 3.4. Association Between PGx Phenotypes and Sertraline PK Outcomes

**Figure 1:**
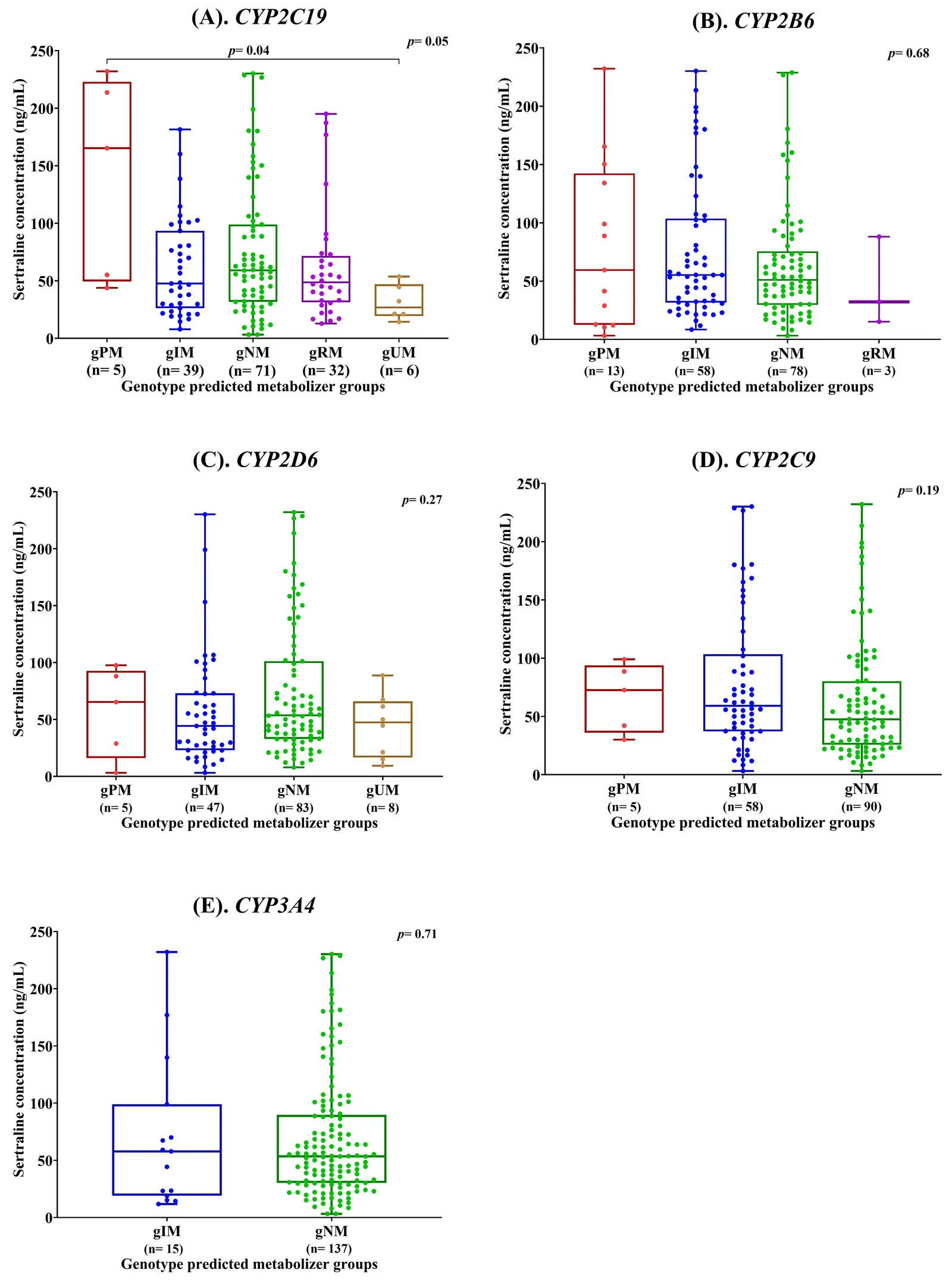
Sertraline plasma concentrations across (**A**) *CYP2C19*, (**B**) *CYP2B6*, (**C**) *CYP2D6*, (**D**) *CYP2C9*, and (**E**) *CYP3A4* genotype-predicted metabolizer groups. Footnotes: Genotype-predicted metabolizer status was indeterminate for 10 patients for *CYP2D6* and for 1 patient for *CYP2B6* and *CYP3A4*. Boxes represent the interquartile range (25th–75th percentiles), horizontal lines within boxes indicate medians, and whiskers extend from the minimum to the maximum observed values. Dots represent individual patient observations. Panels (**A**–**D**) were analyzed using the Kruskal–Wallis test, followed by Dunn’s multiple-comparisons test; panel (**E**) was analyzed using the Mann–Whitney U test. Overall *p*-values are shown in the upper-right corner of each panel, while brackets indicate significant pairwise comparisons with *p*-values. Subgroup sample sizes are provided below the corresponding x-axis labels.

The overall difference in sertraline concentrations across *CYP2C19* genotype-predicted metabolizer groups did not reach statistical significance (*p* = 0.052). The multiple-comparisons test identified a significant difference only between *CYP2C19* gPMs and gUMs (*p* = 0.04), while all other pairwise comparisons were not statistically significant (Figure 1A). In contrast, no significant differences in sertraline concentrations were observed among *CYP2B6*, *CYP2D6*, *CYP2C9*, and *CYP3A4* genotype-predicted metabolizer groups (Figure 1B–E).

**Figure 2:**
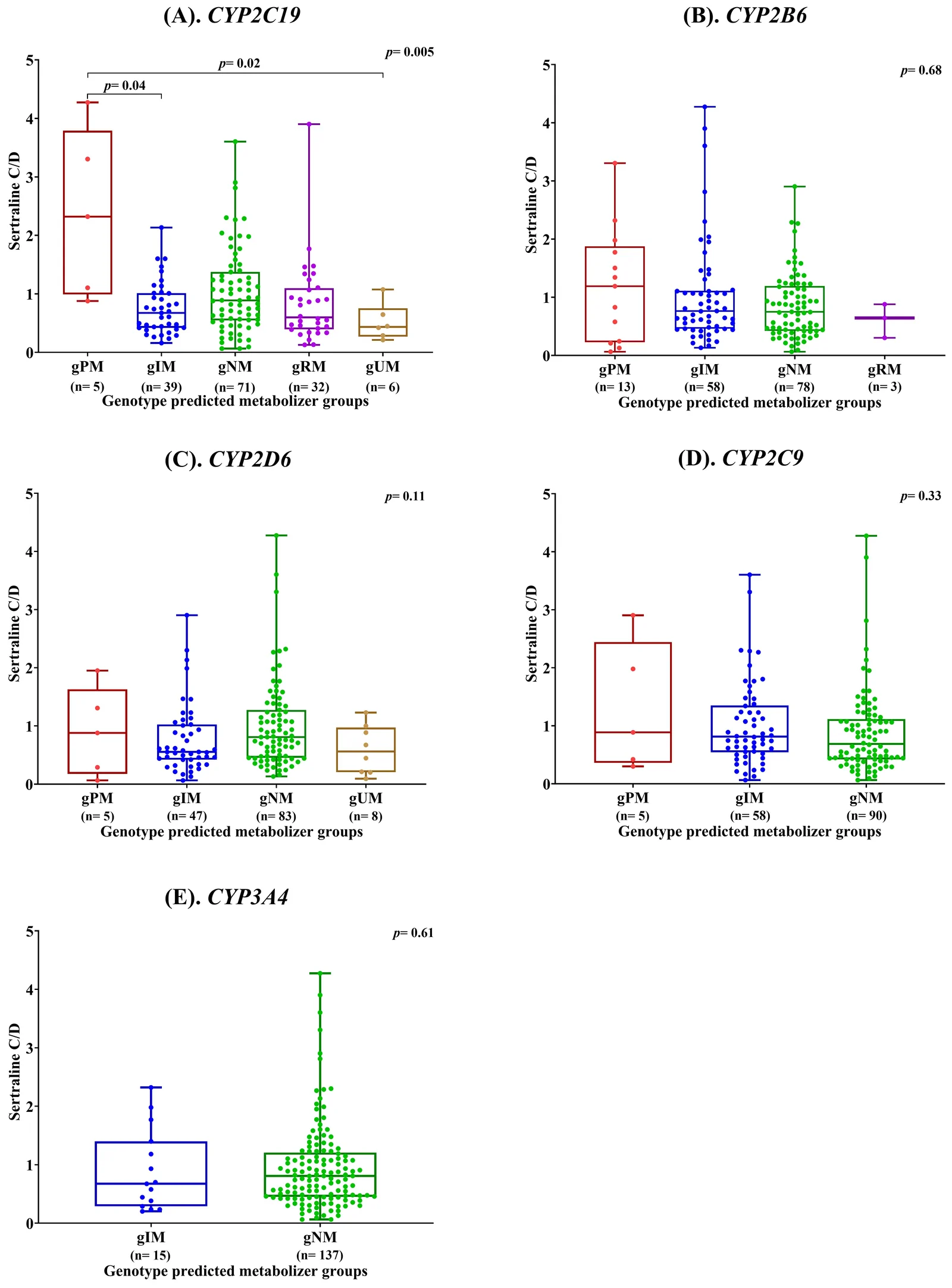
Sertraline concentration-to-dose (C/D) across (**A**) *CYP2C19*, (**B**) *CYP2B6*, (**C**) *CYP2D6*, (**D**) *CYP2C9*, and (**E**) *CYP3A4* genotype-predicted metabolizer groups. Footnotes: Genotype-predicted metabolizer status was indeterminate for 10 patients for *CYP2D6* and for 1 patient for *CYP2B6* and *CYP3A4*. Boxes represent the interquartile range (25th–75th percentiles), horizontal lines within boxes indicate medians, and whiskers extend from the minimum to the maximum observed values. Dots represent individual patient observations. Panels (**A**–**D**) were analyzed using the Kruskal–Wallis test, followed by Dunn’s multiple-comparisons test; panel (**E**) was analyzed using the Mann–Whitney U test. Overall *p*-values are shown in the upper-right corner of each panel, while brackets indicate significant pairwise comparisons with *p*-values. Subgroup sample sizes are provided below the corresponding x-axis labels.

Sertraline C/D differed significantly across *CYP2C19* genotype-predicted metabolizer groups (overall *p* = 0.005). *CYP2C19* gPMs had higher C/D values than gIMs (*p* = 0.04) and gUMs (*p* = 0.02) (Figure 2A). In contrast, no significant differences in sertraline C/D were observed among *CYP2B6*, *CYP2D6*, *CYP2C9*, and *CYP3A4* genotype-predicted metabolizer groups (Figure 2B–E).

**Figure 3:**
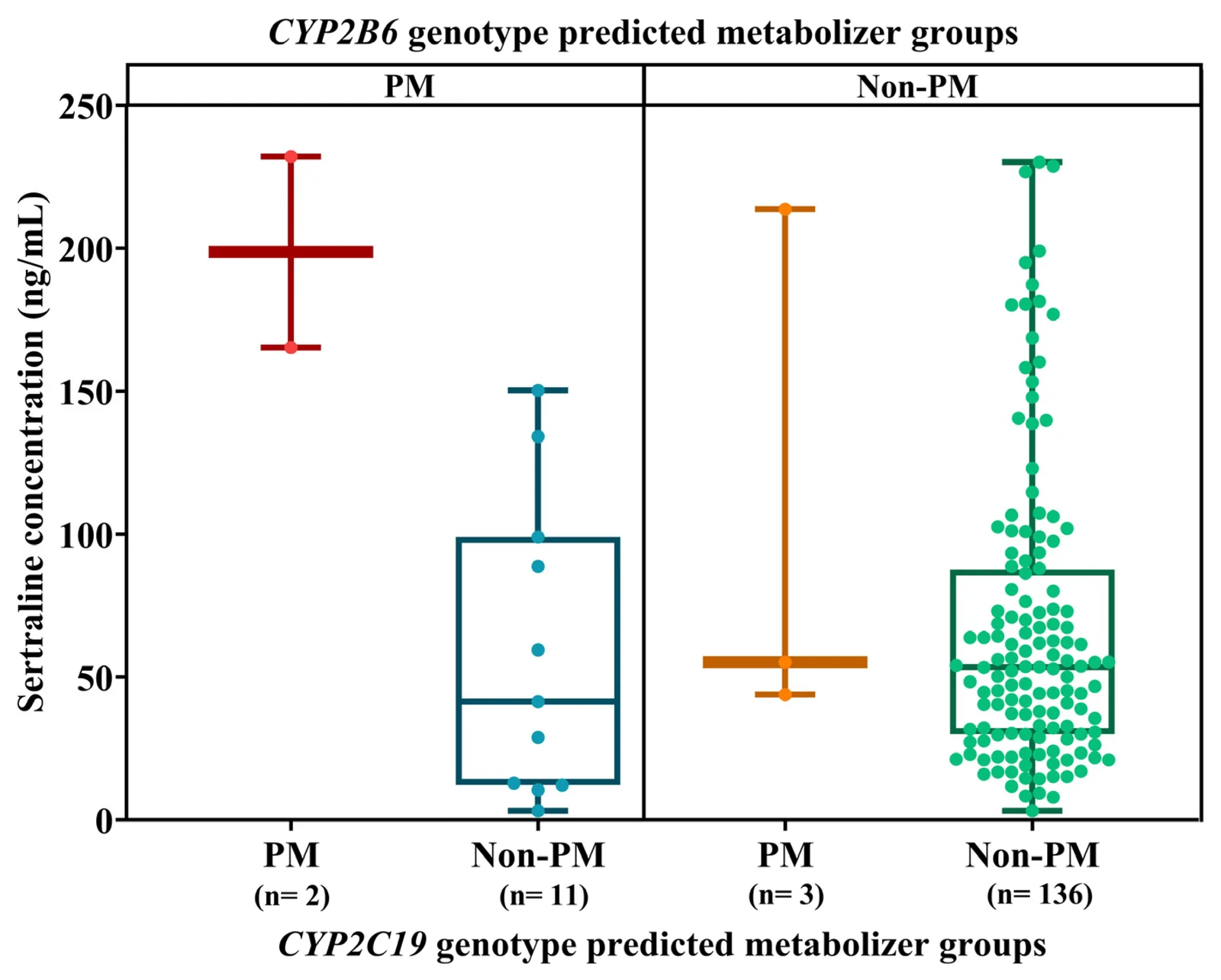
Exploratory descriptive analysis of sertraline plasma concentrations across combined *CYP2C19* and *CYP2B6* genotype-predicted metabolizer groups. Footnote: *CYP2C19* PM and *CYP2B6* PM groups were defined according to CPIC genotype-predicted metabolizer classifications. Non-PM includes gIM, gNM, gRM, and gUM. Boxes represent the interquartile range (25th–75th percentiles), horizontal lines within boxes indicate medians, and whiskers extend from the minimum to the maximum observed values. Dots represent individual patient observations. Subgroup sample sizes are provided below the corresponding x-axis labels. Of the 11 patients with *CYP2B6* gPM status but without *CYP2C19* gPM status, 1, 7, and 3 were *CYP2C19* gIMs, gNMs, and gRMs, respectively. Among the 136 patients who were non-poor metabolizers for both *CYP2B6* and *CYP2C19*, 37, 64, 29, and 6 were *CYP2C19* gIMs, gNMs, gRMs, and gUMs, respectively. Only 1 patient with unavailable *CYP2B6* genotype-predicted metabolizer status was excluded from the combined-gene analysis.

In an exploratory descriptive analysis, the two patients with *CYP2C19* and *CYP2B6* gPM status had sertraline concentrations of 165.3 and 232.1 ng/mL (Figure 3). As this subgroup included only two patients, no formal statistical analyses were performed; therefore, the findings should be considered hypothesis-generating, interpreted with caution, and require confirmation in larger cohorts.

The two patients with both *CYP2C19* and *CYP2B6* gPM status also showed higher sertraline C/D values than the other combined-phenotype groups in the descriptive *CYP2C19-CYP2B6* combined-gene analysis; however, the very small subgroup precluded formal statistical comparison (Supplementary Figure S1).

**Figure 4:**
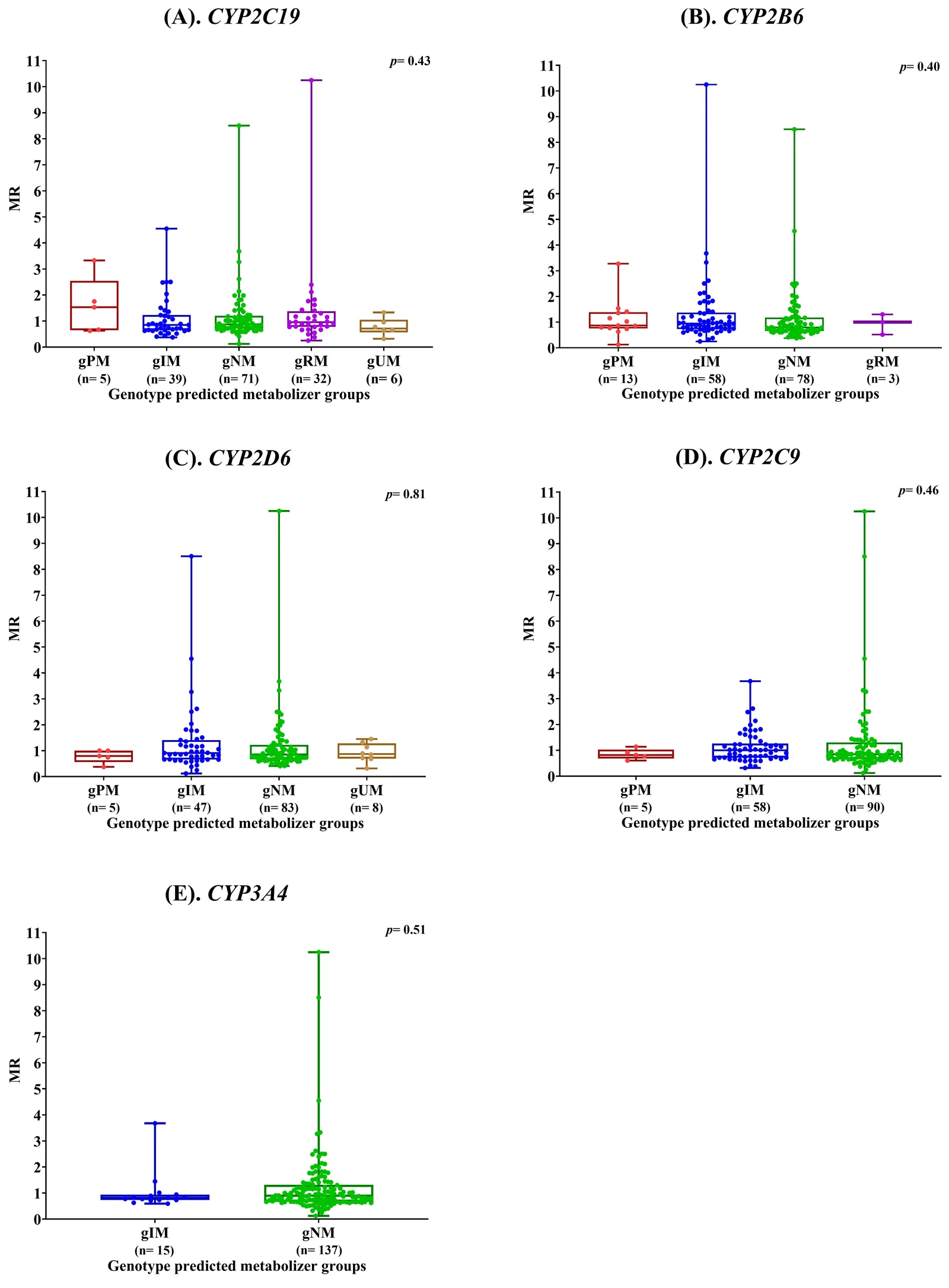
Sertraline-to-norsertraline MR across (**A**) *CYP2C19*, (**B**) *CYP2B6*, (**C**) *CYP2D6*, (**D**) *CYP2C9*, and (**E**) *CYP3A4* genotype-predicted metabolizer groups. Footnotes: Genotype-predicted metabolizer status was indeterminate for 10 patients for *CYP2D6* and for 1 patient for *CYP2B6* and *CYP3A4*. Boxes represent the interquartile range (25th–75th percentiles), horizontal lines within boxes indicate medians, and whiskers extend from the minimum to the maximum observed values. Dots represent individual patient observations. Panels (**A**–**D**) were analyzed using the Kruskal–Wallis test, followed by Dunn’s multiple-comparisons test; panel (**E**) was analyzed using the Mann–Whitney U test. Overall *p*-values are shown in the upper-right corner of each panel, while brackets indicate significant pairwise comparisons with *p*-values. Subgroup sample sizes are provided below the corresponding x-axis labels.

The MR did not differ significantly across genotype-predicted metabolizer groups for *CYP2C19*, *CYP2B6*, *CYP2D6*, *CYP2C9*, or *CYP3A4* (Figure 4).

The LogMR_DN_ also did not differ significantly across genotype-predicted metabolizer groups for *CYP2C19*, *CYP2B6*, *CYP2D6*, *CYP2C9*, or *CYP3A4* (Supplementary Figure S2).

### 3.5. Influence of CYP2C:TG Haplotype According to CYP2C19 Phenotype (Exploratory)

**Figure 5:**
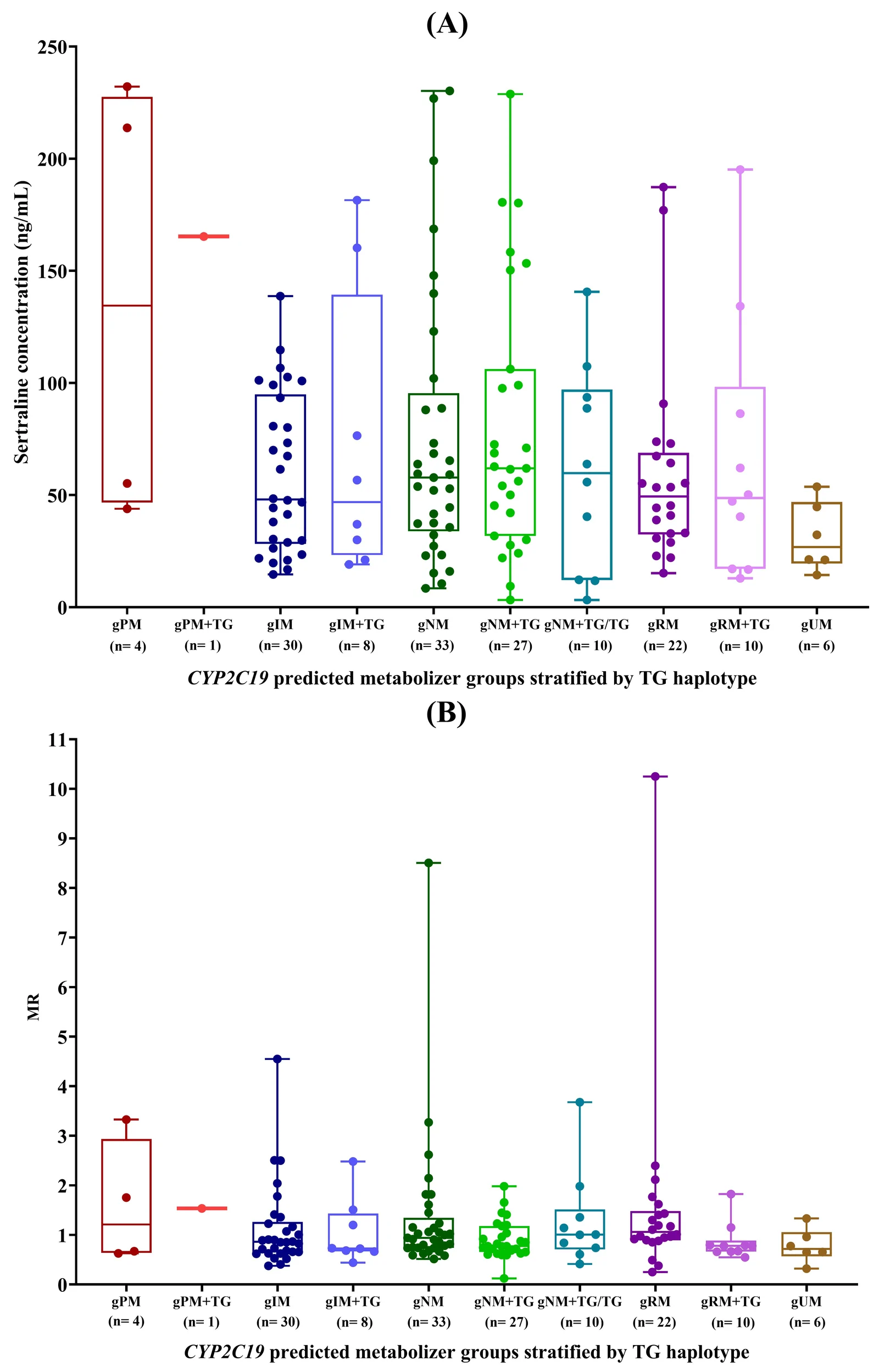
Sertraline concentrations and MR according to *CYP2C:TG* haplotype status within *CYP2C19* genotype-predicted metabolizer groups. (**A**) Sertraline concentration and (**B**) MR. Footnotes: “+TG” denotes carriage of one *CYP2C:TG* haplotype, whereas “+TG/TG” denotes carriage of two copies. *CYP2C19:TG* haplotype status was indeterminate for two patients (one gNM and one gIM), who were excluded from the TG haplotype subgroup analysis. Boxes represent the interquartile range (25th–75th percentiles), horizontal lines within boxes indicate medians, and whiskers extend from the minimum to the maximum observed values. Dots represent individual patient observations. Subgroup sample sizes are provided below the corresponding x-axis labels.

This exploratory descriptive analysis was performed without formal statistical testing due to limited sample sizes after stratification by *CYP2C19* phenotype. Sertraline concentrations were broadly comparable between *CYP2C:TG* haplotype carriers and non-carriers within the *CYP2C19* gIM, gNM, and gRM groups, with substantial overlap in their distributions (Figure 5A). Likewise, MR distributions showed substantial overlap between *TG* haplotype carriers and non-carriers within each *CYP2C19* metabolizer phenotype, showing little evidence of meaningful differences associated with TG haplotype status. (Figure 5B). Given the small subgroup sizes, these observations should be interpreted descriptively and considered exploratory.

Within *CYP2C19* phenotype groups, sertraline C/D and LogMR_DN_ distributions also showed substantial overlap between *CYP2C:TG* haplotype carriers and non-carriers; these analyses were also descriptive due to the small sample sizes of several stratified subgroups (Supplementary Figure S3).

### 3.6. Multivariable Determinants of Sertraline PK Outcomes

In the sertraline concentration model, *CYP2C19* gPM status was associated with a 72.10-ng/mL higher concentration than gNM status (95% CI: 21.36 to 122.84; *p* = 0.01). *CYP2C19* gPM status was also associated with a higher sertraline C/D than gNM status (β = 1.46, 95% CI: 0.85 to 2.08; *p* < 0.001). No other PGx, demographic, clinical, or prescribing predictor was significantly associated with sertraline concentration or C/D. In the MR model, current smoking was associated with a higher MR than non-smoking or former smoking (β = 0.69, 95% CI: 0.11 to 1.26; *p* = 0.02), as shown in Table 3. No predictor was significantly associated with the LogMR_DN_. In the LogMR model, *CYP2C:TG* haplotype carriage was associated with lower LogMR (β = −0.11, 95% CI: −0.21 to −0.00; *p* = 0.04), whereas current smoking was associated with higher LogMR (β = 0.14, 95% CI: 0.02 to 0.26; *p* = 0.03). Increasing age was weakly associated with lower LogMR (β = −0.0034 per year, 95% CI: −0.0067 to −0.00002; *p* = 0.048), as shown in Supplementary Table S6.

After accounting for the number of predictors, based on the adjusted R^2^ values, the multivariable models explained 8% of the variability in sertraline concentration (adjusted R^2^ = 0.08; multiple R^2^ = 0.20; *p* = 0.06), 21% in sertraline C/D (adjusted R^2^ = 0.21; multiple R^2^ = 0.31; *p* < 0.01), 5% in MR (adjusted R^2^ = 0.05; multiple R^2^ = 0.17; *p* = 0.12), and 6% in LogMR (adjusted R^2^ = 0.06; multiple R^2^ = 0.19; *p* = 0.09). The LogMR_DN_ model explained negligible variability after adjustment (adjusted R^2^ ≈ 0.00; multiple R^2^ = 0.11; *p* = 0.67). No meaningful multicollinearity was observed among the included predictors (adjusted generalized variance inflation factor: 1.08–1.34). The Breusch–Pagan (BP) test showed no evidence of heteroscedasticity in the sertraline concentration (BP = 16.26, df = 18, *p* = 0.57), MR (BP = 14.15, df = 18, *p* = 0.72), LogMR_DN_ (BP = 17.33, df = 18, *p* = 0.50), or LogMR (BP = 17.29, df = 18, *p* = 0.50) models as shown in Supplementary Table S7. However, heteroscedasticity was detected in the sertraline C/D model (BP = 37.69, df = 18, *p* < 0.01); therefore, HC3 heteroscedasticity-robust standard errors were used in a sensitivity analysis. In the HC3 sensitivity analysis, no predictor was statistically significantly associated with C/D. *CYP2C19* gPM status showed suggestive but non-significant evidence of higher C/D (β = 1.46, 95% CI: −0.09 to 3.02; *p* = 0.07), as shown in Supplementary Table S8. Visual inspection of the Q–Q plots suggested the most pronounced departure from residual normality in the MR model, particularly in the upper tail. Less-marked tail deviations were observed for the sertraline concentration, C/D, and LogMR models, whereas the LogMR_DN_ residuals followed the reference line more closely, apart from a small number of extreme observations (Figures S4–S13).

## 4. Discussion

The present study provides a comprehensive real-world evaluation of the relative contributions of pharmacogenes including *CYP2C19*, *CYP2B6*, *CYP2D6*, *CYP2C9*, and *CYP3A4*; the *CYP2C:TG* haplotype; and demographic, clinical, and prescribing characteristics to three main sertraline PK outcomes—sertraline plasma concentration, C/D, and MR, along with LogMR_DN_ and LogMR examined as exploratory PK outcomes. *CYP2C19* gPM status was associated with higher sertraline concentrations in the multivariable model. Although *CYP2C19* gPM status was also associated with higher sertraline C/D ratios in the conventional multivariable analysis, this association was not retained after applying HC3 heteroscedasticity-robust standard errors, suggesting that the association should be interpreted with caution. Current smoking was independently associated with higher MR and higher LogMR, whereas *CYP2C:TG* haplotype carriage was associated with lower LogMR. No individual predictor was significantly associated with LogMR_DN_. No independent associations were identified for *CYP2B6*, *CYP2D6*, *CYP3A4*, or *CYP2C9*. Collectively, these findings imply that the factors associated with parent-sertraline exposure may differ from those associated with composite measures of sertraline and norsertraline disposition. Nevertheless, associations between rare metabolizer groups and the metabolic-ratio outcomes should be regarded as exploratory due to limited subgroup sizes, multiple comparisons, and the model’s modest explanatory power.

Despite the involvement of multiple CYP enzymes in sertraline biotransformation, the present findings suggest that *CYP2C19* may influence sertraline exposure in routine clinical practice. These findings are consistent with previous PGx studies identifying *CYP2C19* as a determinant of sertraline PK [11,28] and are further supported by international PGx consortia, which assign *CYP2C19* the highest level of evidence supporting genotype-guided sertraline prescribing (CPIC Level A recommendation; ClinPGx Level 1A evidence) [29]. The multivariable analyses supports an influence of *CYP2C19* status on sertraline exposure, with gPM having relatively higher sertraline concentrations, as supported by several studies [30,31]. Consistent with a Scandinavian study of 1202 patients reporting 2.68-fold higher dose-harmonized sertraline concentrations in *CYP2C19* PMs than in NMs [30], *CYP2C19* gPM status was associated with higher sertraline C/D ratios in our study cohort. Moreover, the estimated increase in sertraline concentration of approximately 72 ng/mL associated with *CYP2C19* gPM status in the multivariable model was notable relative to the therapeutic reference range. However, this estimate was based on only a few *CYP2C19* gPMs, and the study did not assess treatment response or adverse outcomes; therefore, its clinical implications and potential relevance to *CYP2C19*-informed dosing require confirmation in larger cohorts. Although *CYP2C19* gUM status was associated with lower sertraline concentrations and C/D ratios than gNM status, neither association reached statistical significance, possibly reflecting the small number of gUM patients, but was directionally consistent with enhanced *CYP2C19*-mediated metabolism.

*CYP2C19* phenotype was not significantly associated with MR or LogMR_DN_ in either the genotype-group comparisons or the multivariable analyses. Pharmacologically, this is plausible because MR and LogMR_DN_ are composite measures integrating parent-drug exposure, norsertraline formation, and subsequent metabolite disposition, rather than *CYP2C19*-mediated *N*-demethylation alone. Although *CYP2C19* is a clinically important determinant of parent sertraline exposure, no individual CYP enzyme is thought to mediate more than 25–35% of sertraline *N*-demethylation [10]. Furthermore, both sertraline and norsertraline undergo additional biotransformation through multiple parallel oxidative and conjugative pathways involving CYP enzymes, monoamine oxidases, and UDP-glucuronosyltransferases (UGTs) [10]. Consequently, MR and LogMR_DN_ represent composite phenotypic measures of sertraline metabolism to norsertraline and subsequent norsertraline disposition, rather than a specific marker of *CYP2C19*-mediated *N*-demethylation. This likely explains why *CYP2C19* influenced parent sertraline concentrations but not MR and LogMR_DN_. Moreover, variability in both norsertraline formation and its subsequent elimination through multiple metabolic pathways may obscure the contribution of individual enzymes when composite metabolic ratios are used as endpoints.

The current CPIC PGx guidelines for sertraline establish joint guidelines for *CYP2C19* and *CYP2B6* [14], supported by scientific studies demonstrating associations between *CYP2C19* and *CYP2B6* and sertraline concentrations [12,32]. In the present study, no significant differences were observed across *CYP2B6* genotype-predicted metabolizer groups in sertraline concentration, C/D, MR, and LogMR_DN_. This may be because *CYP2B6* is only one of several enzymes involved in sertraline *N*-demethylation [10], with substantial overlap in substrate metabolism among *CYP2C19*, *CYP2D6*, *CYP2C9*, *CYP3A4*, and other metabolic pathways. However, two patients with both *CYP2C19* and *CYP2B6* gPM status had concentrations above the proposed AGNP laboratory alert level of 150 ng/mL; hence, the present data do not establish incremental predictive utility for *CYP2B6*; rather, they support evaluation of combined *CYP2C19*/*CYP2B6* effects in larger cohorts. This analysis was exploratory, and the findings should not be considered sufficient to support clinical application at this stage. These findings align with a study reporting that patients with combined *CYP2C19* and *CYP2B6* PM status had a 2.89-fold higher predicted mean sertraline plasma concentration than *CYP2C19*/*CYP2B6* NMs [13]. Likewise, another study showed that incorporating both *CYP2C19* and *CYP2B6* genotypes into a combinatorial PGx algorithm improved prediction of sertraline blood concentrations compared with single-gene approaches [33]. The overall contribution of other pharmacogenes to sertraline PK appears to be limited in the present study, consistent with previous reports [11,34]. However, because some metabolizer subgroups were represented by relatively few patients, modest genotype-related effects cannot be entirely ruled out.

Descriptive analyses showed substantial overlap in sertraline concentration, C/D, MR, and LogMR_DN_ distributions between *CYP2C:TG* carriers and non-carriers within *CYP2C19* phenotype groups. In the multivariable analyses, *CYP2C:TG* haplotype carriage was not associated with sertraline concentration, C/D, MR, or LogMR_DN_, consistent with previous report [35], but was associated with lower LogMR. The substantial overlap and small sizes of several haplotype-stratified subgroups limit firm conclusions regarding incremental value beyond conventional *CYP2C19* genotype classification. Accordingly, the LogMR association should be regarded as exploratory and warrants further assessments in larger independent cohorts.

The observed genotype-predicted phenotype distributions were generally consistent with those previously reported in populations of European ancestry; however, direct comparisons should be interpreted with caution given the modest sample size, particularly for the less frequent genotypes and metabolizer groups [36,37,38,39,40,41,42,43].

Sertraline PGx dosing recommendations differ across the CPIC and the DPWG. CPIC provides detailed, genotype-specific guidance for both *CYP2C19* and *CYP2B6*, whereas DPWG restricts its recommendations to *CYP2C19* PMs [14,44]. These consortia or regulatory guidelines offer no actionable recommendations for any other genes. Regarding regulatory agencies, the U.S. Food and Drug Administration (FDA) provides no actionable PGx dosing recommendations for sertraline [45], while the Spanish Drug and Health Products Regulatory Agency (AEMPS) notes that although sertraline plasma levels are 50% higher in *CYP2C19* PMs than in RMs, the clinical relevance is unclear; therefore, dose titration should be guided by clinical response [46].

In addition to PGx variability, sertraline PK has been reported to be influenced by non-genetic factors, including smoking, age, eGFR, total protein, AST level, and sex [7,8,34,47]. In the univariable analyses, older age was associated with higher sertraline C/D and lower MR, whereas polypharmacy was associated with higher sertraline concentration and C/D but lower MR and LogMR_DN_. However, the number of concomitant medications, entered as a continuous covariate in the multivariable models, was not independently associated with any sertraline PK outcome, suggesting that the univariable polypharmacy associations may partly reflect broader clinical and prescribing complexity rather than independent medication effects. The weak association between increasing age and lower LogMR in the multivariable model may reflect age-related changes in the disposition of sertraline and norsertraline; however, given its small effect size, this isolated exploratory finding warrants cautious interpretation. Patients with ≥5 diagnoses were also associated with lower MR and LogMR_DN_ in univariable analyses. Among non-genetic factors, current smoking was independently associated with MR and LogMR, but not with LogMR_DN_ in multivariable analysis. Smoking has been reported to induce *CYP2E1* activity [48], one of the enzymes involved in the downstream metabolism of norsertraline [10], and may therefore contribute to the association observed with MR and LogMR, although the quantitative contribution of *CYP2E1* to desmethylsertraline metabolism remains uncertain. These smoking associations should be interpreted cautiously and as exploratory.

Beyond its effects on sertraline PK, *CYP2C19* genetic variation has also been associated with sertraline treatment outcomes, including antidepressant switching and ADRs. The rate of antidepressant switching for sertraline was higher in *CYP2C19* gPMs and gIMs compared to gNMs by 5.8-fold and 2.8-fold, respectively, in a large-scale Chinese Han cohort [49]. A retrospective study of Australian adults reported that, compared with gNMs, gIMs had higher odds of reporting any sertraline side effect (*p* = 0.009), with the strongest associations observed for weight loss, fatigue, and drowsiness [50].

The major limitation of the present study is that, despite comprehensively characterizing the PGx determinants of sertraline PK, it did not assess whether genotype- and exposure-related differences translated into clinically relevant outcomes, including antidepressant response, treatment switching, medication adherence, or ADRs. The genotypes reported for the analyzed genes were determined using a targeted genetic panel. Therefore, it cannot be excluded that some patients may carry additional genetic variants that were not assessed in the present study. Because this was a naturalistic study conducted in routine clinical practice, treatment adherence was assessed by patient self-report and was not objectively verified using pill counts, dispensing records, electronic monitoring, or directly observed dosing. Although samples were collected immediately before the next scheduled dose, consistent adherence to the prescribed regimen and the exact timing of preceding doses could not be confirmed with complete certainty. The combined *CYP2C19*/*CYP2B6* observations may be relevant in selected patients but are not sufficiently precise to justify broader recommendations without replication in larger-sample-sized patient populations. Concomitant treatment is an important consideration in PK analyses because enzyme inhibitors may modify genetically predicted metabolic activity. Strong inhibitor exposure was uncommon, involving only two patients for *CYP2D6* and one patient for *CYP2C19*. Moderate inhibitor exposure was also limited for *CYP2D6* (n = 1) but occurred in 10 patients for *CYP2C19*. Because the exposure categories overlapped and very few patients received strong inhibitors, the corresponding PK outcomes were examined descriptively without formal between-group comparisons (Supplementary Table S2). Consequently, the present data have limited power to quantify inhibitor-related phenoconversion, particularly for strong inhibitors, and do not rule out clinically relevant DDI effects in individual patients. The sertraline C/D multivariable model showed evidence of heteroscedasticity, suggesting that the conventional standard errors, confidence intervals, and *p*-values may be affected by non-constant residual variance. Together with the small numbers in some genotype-predicted metabolizer groups, this finding warrants cautious interpretation of the precision and statistical significance of the C/D model estimates. Additional limitations should also be considered. The study population was predominantly elderly, with a median age of 73 years. Dose selection in this older, multimorbid and polymedicated cohort was likely influenced by several clinical considerations, including polypharmacy, DDIs, tolerability, comorbidity burden and previous treatment response. Because the analyzed PGx results were unavailable to prescribers when sertraline treatment and dose were selected, genotype did not influence prescribing decisions. Extrapolation of these findings to younger populations receiving sertraline should be made with caution. Some metabolizer phenotype groups, particularly gPMs for certain pharmacogenes, were represented by relatively small numbers of patients, which may have limited statistical power to detect modest genotype-related differences. Despite these limitations, the present study provides one of the most comprehensive real-world evaluations of the combined influence of PGx, demographic, clinical, and prescribing determinants on sertraline PK within a clinical PGx implementation setting.

## 5. Conclusions

This real-world cohort study showed that *CYP2C19* gPM status is independently associated with higher sertraline concentrations; although it was also associated with higher C/D in the conventional multivariable analysis, this association was not retained after applying HC3 robust standard errors to account for heteroscedasticity. However, because these estimates were based on a small number of gPMs and clinical outcomes were not evaluated, the findings establish PK relevance but do not independently demonstrate the clinical utility of *CYP2C19*-guided prescribing. Current smoking was independently associated with higher MR and LogMR, although these findings should be considered exploratory and require replication. The exploratory findings showing elevated sertraline concentrations and C/D ratio in patients with both *CYP2C19* and *CYP2B6* gPM status are hypothesis-generating and warrant confirmation in larger cohorts. Overall, the study findings support further prospective evaluation of multigene approaches integrated with PK and their impact on clinical outcomes in larger patient populations. A key strength of this study is the simultaneous evaluation of multiple PGx and non-genetic determinants within a real-world clinical implementation setting, allowing assessment of their relative contributions to sertraline PK variability.

## Figures and Tables

**Table 1:** Analytical performance of the assay: accuracy and precision for sertraline and norsertraline.

Drug	Theoretical Concentration (ng/mL)	Measured Concentration (ng/mL)	Accuracy	Precision (CV, %)
**Sertraline**	76.92	71.32	92.73	4.1
	19.23	20.24	105.29	4.66
	4.81	5.14	106.89	1.3
**Norsertraline**	76.92	72.59	94.37	3.43
	19.23	20.85	103.99	10.25
	4.81	6.30	131	6.3

**Table 2:** Univariate association of demographic, clinical, and prescribing characteristics with sertraline concentration, C/D, and MR.

Variable	Group	N (%)	Sertraline Concentration Median (Q1, Q3)	*p*-Value	Sertraline C/D Median (Q1, Q3)	*p*-Value	MR Median (Q1, Q3)	*p*-Value
Age	≥65 years	100 (65.4)	55.6 (31.9, 93.6)	0.32	0.9 (0.5, 1.3)	**0.02**	0.8 (0.7, 1.1)	**0.0004**
	<65 years	53 (34.6)	50.2 (25.7, 72.1)		0.6 (0.4, 1.1)		1.1 (0.8, 1.8)	
Sex	Male	40 (26.1)	46.3 (28.5, 94.5)	0.48	0.7 (0.4, 1.1)	0.25	0.8 (0.7, 1.3)	0.60
	Female	113 (73.9)	55.2 (30.3, 89.7)		0.8 (0.4, 1.2)		0.9 (0.7, 1.2)	
BMI (kg/m^2^)	<25	41 (26.8)	53.7 (30.6, 78.6)	0.89	0.8 (0.5, 1.3)	0.54	0.9 (0.7, 1.6)	0.55
	≥25	112 (73.2)	53.7 (29.1, 93.6)		0.8 (0.4, 1.2)		0.9 (0.7, 1.2)	
Smoking	Current smoking	25 (16.3)	45.3 (22.8, 70.3)	0.28	0.6 (0.3, 1.1)	0.15	1.0 (0.8, 1.8)	0.07
	Non-smoker/Former smoker	128 (83.7)	54.0 (30.5, 92.7)		0.8 (0.5, 1.2)		0.9 (0.7, 1.2)	
Polypharmacy	Yes	138 (90.2)	54.7 (31.6, 93.5)	**0.03**	**0.8 (0.5, 1.2)**	**0.04**	0.9 (0.7, 1.2)	**0.01**
	No	15 (9.8)	27.3 (15.2, 56.7)		0.4 (0.3, 0.9)		1.3 (0.9, 2.6)	
Number of diagnoses	≥5	114 (74.5)	53.6 (31.6, 88.7)	0.83	0.8 (0.5, 1.2)	0.45	0.8 (0.7, 1.1)	**0.0001**
	<5	39 (25.5)	54.1 (23.5, 102.6)		0.7 (0.3, 1.1)		1.2 (0.8, 2.1)	
ALT (IU/L)	>40	7 (5.8)	38.0 (29.8, 44.3)	0.46	0.6 (0.3, 0.9)	0.43	1.0 (0.7, 2.5)	0.59
	≤40	113 (94.2)	55.2 (26.8, 83.5)		0.8 (0.4, 1.2)		0.9 (0.7, 1.3)	
AST (IU/L)	>40	2 (1.9)	110.7 (44.3, 177.0)	0.46	1.3 (0.9, 1.8)	0.31	1.0 (1.0, 1.0)	0.78
	≤40	105 (98.1)	53.8 (27.5, 83.5)		0.8 (0.4, 1.2)		0.9 (0.7, 1.4)	
GGT (IU/L)	>50	15 (15.2)	62.7 (23.0, 106.7)	0.71	0.7 (0.5, 1.5)	0.55	1.0 (0.7, 1.1)	0.62
	≤50	84 (84.8)	53.8 (30.5, 75.8)		0.8 (0.4, 1.2)		0.9 (0.7, 1.2)	
ALP (IU/L)	>120	8 (11.6)	58.4 (22.3, 138.4)	0.83	1.1 (0.4, 1.4)	0.54	1.2 (0.7, 1.7)	0.34
	≤120	61 (88.4)	53.8 (32.0, 89.7)		0.8 (0.5, 1.3)		0.9 (0.7, 1.2)	
Urea (mg/dL)	>40	50 (41.7)	50.6 (22.0, 86.9)	0.40	0.8 (0.4, 1.1)	0.18	0.9 (0.7, 1.2)	0.20
	≤40	70 (58.3)	55.3 (30.7, 92.8)		0.8 (0.5, 1.3)		0.9 (0.7, 1.5)	

Footnotes: Data were unavailable for 33 patients for ALT, 46 for AST, 54 for GGT, 84 for ALP, and 33 for urea. Between-group comparisons were performed using the Mann–Whitney test. Bold values indicate statistical significance (*p* < 0.05).

**Table 3:** Multivariable linear regression analysis of PGx, demographic, clinical, and prescribing determinants of sertraline concentrations, sertraline C/D, and MR (N = 140).

Predictors	Sertraline Concentration (ng/mL)		Sertraline C/D [(ng/mL)/(mg/Day)]		MR	
	β (95% CI)	*p*-Value	β (95% CI)	*p*-Value	β (95% CI)	*p*-Value
*CYP2D6* phenotype (Ref= gNM)						
gIM	−15.62 (−35.47, 4.24)	0.12	−0.19 (−0.43, 0.05)	0.13	0.02 (−0.44, 0.47)	0.95
gPM	−15.48 (−64.30, 33.34)	0.53	−0.06 (−0.65, 0.53)	0.84	−0.35 (−1.46, 0.76)	0.54
gUM	−28.33 (−68.67, 12.00)	0.17	−0.42 (−0.91, 0.07)	0.09	−0.19 (−1.11, 0.73)	0.69
*CYP2C19* phenotype (Ref = gNM)						
gIM	−10.96 (−34.50, 12.57)	0.36	−0.16 (−0.44, 0.13)	0.28	−0.38 (−0.92, 0.15)	0.16
gPM	72.10 (21.36, 122.84)	**0.01**	1.46 (0.85, 2.08)	**<0.001**	0.17 (−0.98, 1.33)	0.77
gRM	−14.82 (−39.12, 9.48)	0.23	−0.23 (−0.52, 0.07)	0.13	−0.18 (−0.73, 0.38)	0.53
gUM	−36.62 (−82.95, 9.71)	0.12	−0.35 (−0.91, 0.21)	0.22	−0.73 (−1.79, 0.33)	0.17
*CYP2B6* phenotype (Ref = gNM)						
gIM	7.23 (−12.26, 26.71)	0.46	0.10 (−0.14, 0.33)	0.42	0.20 (−0.25, 0.64)	0.38
gPM	0.02 (−32.22, 32.26)	1.00	0.02 (−0.37, 0.41)	0.91	0.04 (−0.69, 0.78)	0.91
gRM	−12.36 (−76.62, 51.90)	0.70	−0.20 (−0.98, 0.58)	0.61	−0.30 (−1.76, 1.17)	0.69
*CYP2C9* phenotype (Ref = gNM)						
gIM	18.41 (−1.92, 38.75)	0.08	0.16 (−0.09, 0.41)	0.20	−0.09 (−0.55, 0.38)	0.71
gPM	16.91 (−35.28, 69.10)	0.52	0.53 (−0.10, 1.16)	0.10	0.17 (−1.02, 1.36)	0.78
*CYP3A4* phenotype (Ref = gNM)						
gIM	0.17 (−30.22, 30.55)	0.99	−0.08 (−0.45, 0.29)	0.68	−0.56 (−1.26, 0.13)	0.11
*CYP2C:TG* haplotype present (Ref = absent)	−7.02 (−27.98, 13.95)	0.51	−0.03 (−0.29, 0.22)	0.80	−0.43 (−0.91, 0.05)	0.08
Female sex (Ref = male)	−8.15 (−29.21, 12.92)	0.45	−0.02 (−0.28, 0.23)	0.85	−0.27 (−0.75, 0.21)	0.27
Age (per year)	0.11 (−0.58, 0.80)	0.75	0.01 (−0.00, 0.01)	0.23	−0.01 (−0.03, 0.00)	0.09
Current smoker (Ref = non/ex-smoker)	2.95 (−22.36, 28.26)	0.82	0.02 (−0.29, 0.33)	0.91	0.69 (0.11, 1.26)	**0.02**
Number of drugs	1.74 (−0.77, 4.25)	0.17	0.03 (−0.01, 0.06)	0.10	−0.03 (−0.09, 0.02)	0.24

Footnotes: Values are β coefficients with 95% confidence intervals from multivariable linear regression models. All predictors presented were entered simultaneously into each model. Reference categories were gNM for genotype-predicted metabolizer phenotypes, absence of the *CYP2C:TG* haplotype, male sex, and non-smoking/former smoking. Age and number of drugs were entered as continuous variables. Bold values indicate statistical significance (*p* < 0.05).

## Data Availability

The raw data supporting the conclusions of this article will be made available by the authors upon request.

## Supplementary Materials

The following supporting information can be downloaded at https://www.mdpi.com/article/10.3390/pharmaceutics18091133/s1, Table S1: *CYP2D6*, *CYP2C9*, *CYP2C19*, *CYP2B6* and *CYP3A4* variants and their corresponding Taqman^®^ assays (ThermoFisher) utilized for Real-Time PCR genotyping; Table S2: Sertraline PK outcomes according to concomitant *CYP2D6* and *CYP2C19* inhibitor exposure; Table S3: Univariate association of demographic, clinical, and prescribing characteristics with LogMR_DN_; Table S4: *CYP2D6*, *CYP2C9*, *CYP2C19*, *CYP2B6* and *CYP3A4* allele frequency; Table S5: *CYP2D6*, *CYP2C9*, *CYP2C19*, *CYP2B6* and *CYP3A4* genotype frequency; Table S6: Multivariable linear regression analysis of PGx, demographic, clinical, and prescribing determinants of LogMR_DN_ and LogMR (N = 140); Table S7: Breusch–Pagan tests for heteroscedasticity in the multivariable models; Table S8: Sensitivity analysis of the multivariable linear regression model for sertraline concentration-to-dose (C/D) ratio using HC3 heteroscedasticity-robust standard errors (N = 140); Figure S1: Sertraline concentration-to-dose (C/D) ratios across combined *CYP2C19* and *CYP2B6* genotype-predicted metabolizer groups; Figure S2: LogMR_DN_ across (A) *CYP2C19*, (B) *CYP2B6*, (C) *CYP2D6*, (D) *CYP2C9*, and (E) *CYP3A4* genotype-predicted metabolizer groups; Figure S3: (A) Sertraline concentration-to-dose (C/D) ratio and (B) LogMR_DN_ are presented across *CYP2C19* genotype-predicted metabolizer groups stratified by *CYP2C:TG* haplotype status; Figure S4: Residuals vs. fitted values for the sertraline concentration model; Figure S5: Normal Q-Q plot of standardized residuals for the sertraline concentration model; Figure S6: Residuals vs. fitted values for the sertraline concentration-to-dose ratio (C/D) model; Figure S7: Normal Q-Q plot of standardized residuals for the sertraline concentration-to-dose ratio (C/D) model; Figure S8: Residuals vs. fitted values for the metabolic ratio (MR) model; Figure S9: Normal Q-Q plot of standardized residuals for the metabolic ratio (MR) model; Figure S10: Residuals vs. fitted values for the LogMR_DN_ model; Figure S11: Normal Q-Q plot of standardized residuals for the LogMR_DN_ model; Figure S12: Residuals vs. fitted values for the LogMR model; Figure S13: Normal Q-Q plot of standardized residuals for the LogMR model.

## Abbreviations

The following abbreviations are used in this manuscript:

SSRI	Selective serotonin reuptake inhibitor
DDIs	Drug–drug interactions
TDM	Therapeutic drug monitoring
AGNP	Arbeitsgemeinschaft für Neuropsychopharmakologie und Pharmakopsychiatrie
PK	Pharmacokinetics
PGx	Pharmacogenetics
ADRs	Adverse drug reactions
C/D	Concentration-to-dose ratio
MR	Sertraline-to-norsertraline metabolic ratio
MR_DN_	Metabolic ratio dose-normalized
LogMR	Log_10_-transformed sertraline/norsertraline metabolic ratio
LogMR_DN_	Log_10_-transformed dose-normalized sertraline/norsertraline metabolic ratio
BMI	Body mass index
PCR	Polymerase chain reaction
LLOQ	Lower limit of quantification
BP	Breusch–Pagan
gUM	Genotype-predicted ultrarapid metabolizers
gIM	Genotype-predicted intermediate metabolizers
gPM	Genotype-predicted poor metabolizers
gNM	Genotype-predicted normal metabolizers
gRM	Genotype-predicted rapid metabolizers
CPIC	Clinical Pharmacogenetics Implementation Consortium
DPWG	Dutch Pharmacogenetics Working Group
ESI	Electrospray ionization
ALT	Alanine aminotransferase
AST	Aspartate aminotransferase
GGT	Gamma-glutamyl transferase
ALP	Alkaline phosphatase
UGTs	UDP-glucuronosyltransferases
