# Supplementary material for: Pharmacogenetic, Clinical, Prescribing, and Demographic Predictors of Sertraline Pharmacokinetics
Source: Pharmaceutics. 2026 Sep 9;18(9):1133. doi: 10.3390/pharmaceutics18091133 (PMC13610722; doi:10.3390/pharmaceutics18091133)

Supplementary File S1

Table S1. *CYP2D6, CYP2C9*, *CYP2C19, CYP2B6* and *CYP3A4* variants and their corresponding Taqman® assays *(*ThermoFisher*)* utilized for Real-Time PCR genotyping

Gene	Alleles	rsID	Nucleotide change	Activity score	Allele functional status	Taqman® assay ID
CYP2D6	**2*	rs16947	2851C>T	1	Normal	C__27102425_10
		rs1135840	4181G>C			C__27102414_10
	**3*	rs35742686	2550delA	0	None	C__32407232_50
	**4*	rs3892097	1847G>A	0	None	C__27102431_D0
	**5*	NA	Complete gene deletion		No function	
	**6*	rs5030655	1707T>del	0	None	C__32407243_20
	**9*	rs5030656	2616delAAG	0.25	Decreased	C__32407229_60
	**10*	rs1065852	100C>T	0.25	Decreased	C__11484460_40
		rs1135840	4181G>C			C__27102414_10
	**17*	rs28371706	1022C>T	0.5	Decreased	C___2222771_A0
		rs16947	2851C>T			C__27102425_10
		rs1135840	4181G>C			C__27102414_10
	**35*	rs769258	31G>A	1	Normal	C_27102444_F0
		rs16947	2851C>T			C__27102425_10
		rs1135840	4181G>C			C__27102414_10
	**41*	rs28371725	2989G>A	0.25	Decreased	C__34816116_20
		rs16947	2851C>T			C__27102425_10
		rs1135840	4181G>C			C__27102414_10
	CNV	NA	Exon 9		Increased	Hs00010001_cn
	CNV	NA	Intron 6		Increased	Hs04502394_cn
	CNV	NA	Intron 6		Increased	Hs04502391_cn
	CNV	NA	Intron 2		Increased	Hs04083572_cn
CYP2C9	**2*	rs1799853	3608C>T	0.5	Decreased	C_25625805_10
	**3*	rs1057910	42614A>C	0	None	C_27104892_10
	**5*	rs28371686	42619C>G	0.5	Decreased	C_27859817_40
	**6*	rs9332131	10601delA	0	None	C__32287221_20
	**8*	rs7900194	3627G>A	0.5	Decreased	C__25625804_10
CYP2C19	**2*	rs4244285	19154G>A		None	C__25986767_70
	**3*	rs4986893	17948G>A		None	C__27861809_10
		rs3758581	80161A>G		Normal	
	**4*	rs28399504	1A>G		None	C__30634136_10

		rs3758581	80161A>G	Normal	
	**5*	rs56337013	90033C>T	None	C__27861810_10
		rs3758581	80161A>G	Normal	
	**17*	rs12248560	-806C>T	Increased	C____469857_10
CYP2B6	**4*	rs2279343	18053A>G	Increased	C__30634238_F0
	**5*	rs3211371	25505C>T	Normal	C__30634242_40
	**6*	rs3745274	15631G>T	Decreased	C___7817765_60
		rs2279343	18053A>G	Increased	C__30634238_F0
	**7*	rs3211371	25505C>T	Decreased	C_30634242_40
		rs3745274	15631G>T	Decreased	C___7817765_60
		rs2279343	18053A>G	Increased	C__30634238_F0
	**9*	rs3745274	15631G>T	Decreased	C___7817765_C0
CYP3A4	**22*	rs35599367	15389C>T	Decreased	C__59013445_10

Table S2. Sertraline PK outcomes according to concomitant CYP2D6 and CYP2C19 inhibitor exposure

Enzyme	Inhibitor strength	N (%)	Sertraline concentration median (Q1, Q3)	Sertraline C/D median (Q1, Q3)	MR median (Q1, Q3)	Log MR_{DN} median (Q1, Q3)
CYP2D6	Strong	2 (1.3)	39.6 (26.3, 52.9)	0.7 (0.3, 1.1)	0.8 (0.8, 0.9)	-1.9 (-2.1, -1.8)
	Moderate	1 (0.7)	80.7	0.8	0.6	-2.2
	Weak	8 (5.4)	46.5 (31.8, 54.8)	0.5 (0.3, 0.7)	0.9 (0.7, 1.1)	-2.0 (-2.1, -1.9)
	No inhibitor	139 (93.3)	53.8 (28.9, 93.6)	0.8 (0.4, 1.2)	0.9 (0.7, 1.3)	-1.9 (-2.1, -1.7)
CYP2C19	Strong	1 (0.7)	47.2	0.5	0.5	-2.3
	Moderate	10 (6.7)	77.3 (34, 111.1)	1 (0.3, 2.2)	0.9 (0.8, 1.0)	-1.9 (-2.0, -1.8)
	Weak	65 (43.6)	50.2 (30.1, 98.3)	0.9 (0.5, 1.4)	0.9 (0.7, 1.1)	-1.9 (-2.1, -1.7)
	No inhibitor	84 (56.4)	53.9 (28.9, 73.3)	0.7 (0.4, 1.1)	0.9 (0.7, 1.4)	-1.9 (-2.1, -1.7)

Footnotes: Percentages were calculated from the 149 patients with available concomitant medication information; data were unavailable for 4 patients. Inhibitor strength was classified according to Flockhart's Drug Interactions Table. Exposure categories were not mutually exclusive: one patient received both strong and weak CYP2D6 inhibitors; all 10 patients receiving a moderate CYP2C19 inhibitor also received a weak CYP2C19 inhibitor; and the patient receiving a strong CYP2C19 inhibitor also received a weak CYP2C19 inhibitor. "No inhibitor" indicates no exposure to a strong, moderate, or weak inhibitor of the corresponding enzyme. For the CYP2D6 moderate-inhibitor and CYP2C19 strong-inhibitor categories (n = 1), the individual value is presented without quartiles. For groups with n = 2, the median and the two observed values are reported. Owing to overlapping exposure categories and small subgroup sizes, the findings are presented descriptively rather than with formal statistical comparisons.

Table S3. Univariate association of demographic, clinical, and prescribing characteristics with $LogMR_{DN}$

Variable	Group	N (%)	$LogMR_{DN}$ median (Q1, Q3)	*p*-value
Age	≥ 65 years	100 (65.4)	-1.9 (-2.1, -1.7)	0.13
	< 65 years	53 (34.6)	-1.9 (-2.1, -1.5)	
Sex	Male	40 (26.1)	-1.9 (-2.1, -1.8)	0.35
	Female	113 (73.9)	-1.9 (-2.1, -1.7)	
BMI (kg/m^2)	< 25	41 (26.8)	-1.9 (-2.0, -1.6)	0.18
	≥ 25	112 (73.2)	-1.9 (-2.1, -1.7)	
Smoking	Current smoking	25 (16.3)	-1.8 (-2.0, -1.6)	0.14
	Non-smoker/Former smoker	128 (83.7)	-1.9 (-2.1, -1.7)	
Polypharmacy	Yes	138 (90.2)	-1.9 (-2.1, -1.7)	**0.04**
	No	15 (9.8)	-1.7 (-2.0, -1.3)	
Number of diagnoses	≥ 5	114 (74.5)	-1.9 (-2.1, -1.7)	**0.01**
	< 5	39 (25.5)	-1.8 (-2.0, -1.5)	
ALT (IU/L)	> 40	7 (5.8)	-1.9 (-2.0, -1.3)	0.67
	≤ 40	113 (94.2)	-1.9 (-2.1, -1.7)	
AST (IU/L)	> 40	2 (1.9)	-1.9 (-2.0, -1.7)	0.95
	≤ 40	105 (98.1)	-1.9 (-2.1, -1.7)	
GGT (IU/L)	> 50	15 (15.2)	-1.8 (-2.0, -1.7)	0.42
	≤ 50	84 (84.8)	-1.9 (-2.1, -1.7)	
ALP (IU/L)	> 120	8 (11.6)	-1.8 (-1.9, -1.7)	0.23
	≤ 120	61 (88.4)	-1.9 (-2.1, -1.7)	
Urea (mg/dL)	> 40	50 (41.7)	-1.9 (-2.1, -1.7)	0.18
	≤ 40	70 (58.3)	-1.8 (-2.0, -1.6)	

Footnotes: Data were unavailable for 33 patients for ALT, 46 for AST, 54 for GGT, 84 for ALP, and 33 for urea. Between-group comparisons were performed using Welch's t-test or the Mann–Whitney test, as appropriate according to data distribution. Bold values indicate statistical significance ($p < 0.05$).

Table S4. *CYP2D6, CYP2C9, CYP2C19, CYP2B6* and *CYP3A4* allele frequency

***CYP2D6* alleles**	**N**	**%**	***CYP2C19* alleles**	**N**	**%**
***1**	100	33.8	***1**	199	65
***2**	51	17.2	***2**	46	15
***3**	4	1.4	***4**	3	1
***4**	42	14.2	***17**	58	19
***5**	7	2.4			
***6**	1	0.3	***CYP2B6* alleles**	**N**	**%**
***9**	8	2.7	***1**	195	63.7
***10**	15	5.1	***4**	4	1.3
***17**	1	0.3	***5**	21	6.9
***29**	1	0.3	***6**	76	24.8
***35**	13	4.4	***7**	8	2.6
***41**	31	10.5	**ND**	2	0.7
***1x2**	7	2.4			
***2x2**	4	1.4	***CYP3A4* alleles**	**N**	**%**
***9x2**	1	0.3	***1**	289	95.1
**ND**	10	3.4	***22**	15	4.9

***CYP2C9* alleles**	**N**	**%**
***1**	235	76.8
***2**	48	15.7
***3**	23	7.5

Footnote: ND = Not defined

Table S5. *CYP2D6, CYP2C9, CYP2C19, CYP2B6* and *CYP3A4* genotype frequency

***CYP2D6* genotype**	**N**	**%**	***CYP2C9* genotype**	**N**	**%**
***1/*1**	18	11.8	*1/*1	89	58.2
***1/*17**	1	0.7	*1/*2	40	26.1
***1/*2**	20	13.1	*1/*3	17	11.1
***1/*29**	1	0.7	*2/*2	2	1.3
***1/*3**	2	1.3	*2/*3	4	2.6
***1/*35**	4	2.6	*3/*3	1	0.7
***1/*4**	11	7.2			
***1/*41**	17	11.1	***CYP2C19* genotype**	**N**	**%**
***1/*5**	5	3.3	*1/*1	71	46.4
***1/*9**	3	2	*1/*17	32	20.9
***10/*10**	1	0.7	*1/*2	24	15.7
***1x2/*2**	6	3.9	*1/*4	1	0.7
***1x2/*41**	1	0.7	*17/*17	6	3.9
***2/*10**	1	0.7	*2/*17	13	8.5
***2/*2**	4	2.6	*2/*2	4	2.6
***2/*35**	2	1.3	*2/*4	1	0.7
***2/*4**	6	3.9	*4/*17	1	0.7
***2/*41**	3	2			
***2/*9**	3	2	***CYP2B6* genotype**	**N**	**%**
***2x2/*2**	1	0.7	*1/*1	60	39.2
***2x2/*35**	1	0.7	*1/*4	1	0.7
***2x2/*4**	1	0.7	*1/*5	17	11.1
***2x2/*41**	1	0.7	*1/*6	49	32
***3/*35**	1	0.7	*1/*7	8	5.2
***3/*41**	1	0.7	*4/*5	2	1.3
***35/*41**	1	0.7	*4/*6	1	0.7
***4/*10**	12	7.8	*5/*5	1	0.7
***4/*4**	4	2.6	*6/*6	13	8.5
***4/*41**	3	2	ND	1	0.7
***4/*6**	1	0.7			
***41/*41**	2	1.3	***CYP3A4* genotype**	**N**	**%**
***5/*35**	2	1.3	*1/*1	137	90.1
***9/*35**	2	1.3	*1/*22	15	9.9
***9x2/*2**	1	0.7			
**ND**	10	6.5			

Footnote: ND = Not defined

Table S6. Multivariable linear regression analysis of PGx, demographic, clinical, and prescribing determinants of $LogMR_{DN}$ and LogMR (N = 140).

**Predictors**	**$LogMR_{DN}$**		**LogMR**	
	**β (95% CI)**	***p*-value**	**β (95% CI)**	***p*-value**
CYP2D6 phenotype (Ref= gNM)				
gIM	0.02 (-0.11, 0.14)	0.81	-0.01 (-0.11, 0.09)	0.84
gPM	-0.10 (-0.40, 0.21)	0.53	-0.12 (-0.35, 0.12)	0.33
gUM	-0.11 (-0.36, 0.14)	0.39	-0.04 (-0.24, 0.15)	0.65
CYP2C19 phenotype (Ref = gNM)				
gIM	-0.10 (-0.25, 0.04)	0.17	-0.07 (-0.18, 0.04)	0.23
gPM	0.15 (-0.16, 0.47)	0.34	0.08 (-0.17, 0.33)	0.52
gRM	-0.11 (-0.27, 0.04)	0.14	-0.06 (-0.18, 0.06)	0.33
gUM	-0.13 (-0.42, 0.16)	0.38	-0.20 (-0.42, 0.03)	0.09
CYP2B6 phenotype (Ref = gNM)				
gIM	0.04 (-0.08, 0.16)	0.53	0.05 (-0.04, 0.14)	0.30
gPM	0.04 (-0.16, 0.24)	0.72	-0.00 (-0.16, 0.15)	0.97
gRM	-0.00 (-0.40, 0.40)	1.00	-0.03 (-0.34, 0.29)	0.86
CYP2C9 phenotype (Ref = gNM)				
gIM	-0.00 (-0.13, 0.12)	0.95	0.03 (-0.07, 0.13)	0.55
gPM	0.04 (-0.29, 0.36)	0.82	0.08 (-0.18, 0.33)	0.56
CYP3A4 phenotype (Ref = gNM)				
gIM	-0.10 (-0.29, 0.08)	0.27	-0.11 (-0.26, 0.04)	0.14
CYP2C:TG haplotype present (Ref = absent)	-0.09 (-0.22, 0.04)	0.18	-0.11 (-0.21, -0.00)	**0.04**
Female sex (Ref = male)	0.01 (-0.12, 0.14)	0.90	-0.03 (-0.13, 0.08)	0.61
Age (per year)	-0.00 (-0.01, 0.00)	0.58	−0.0034 (−0.0067, −0.00002)	**0.048**
Current smoker (Ref = non/ex-smoker)	0.14 (-0.01, 0.30)	0.07	0.14 (0.02, 0.26)	**0.03**
Number of drugs	-0.01 (-0.02, 0.01)	0.38	−0.0038 (−0.0159, 0.0085)	0.54

*Footnotes: Values are β coefficients with 95% confidence intervals from multivariable linear regression models. All predictors presented were entered simultaneously into each model. Reference categories were gNM for genotype-predicted metabolizer phenotypes, absence of the *CYP2C:TG* haplotype, male sex, and non-smoking/former smoking. Age and number of drugs were entered as continuous variables.

Table S7. Breusch–Pagan tests for heteroscedasticity in the multivariable models.

Pharmacokinetic outcome model	BP statistic	df	*p*-value
Sertraline concentration	16.26	18	0.57
Sertraline C/D	37.69	18	<0.01
MR	14.15	18	0.72
$LogMR_{DN}$	17.33	18	0.5
LogMR	17.29	18	0.5

Abbreviations: BP = Breusch-Pagan, C/D= sertraline plasma concentration-to-daily-dose ratio, df= degrees of freedom, LogMR= log_{10}-transformed sertraline-to-norsertraline metabolic ratio, $LogMR_{DN}$= log_{10}-transformed dose-normalized sertraline-to-norsertraline metabolic ratio, MR= sertraline-to-norsertraline metabolic ratio

Table S8. Sensitivity analysis of the multivariable linear regression model for sertraline concentration-to-dose (C/D) ratio using HC3 heteroscedasticity-robust standard errors (N =140)

**Predictors**	**β (95% CI)**	***p*-value**
CYP2D6 phenotype (Ref = gNM)		
gIM	−0.19 (−0.42, 0.05)	0.12
gPM	−0.06 (−0.92, 0.80)	0.89
gUM	−0.42 (−0.85, 0.01)	0.06
CYP2C19 phenotype (Ref = gNM)		
gIM	−0.16 (−0.40, 0.09)	0.21
gPM	1.46 (−0.09, 3.02)	0.07
gRM	−0.23 (−0.49, 0.04)	0.09
gUM	−0.35 (−0.78, 0.08)	0.11
CYP2B6 phenotype (Ref = gNM)		
gIM	0.10 (−0.16, 0.35)	0.45
gPM	0.02 (−0.49, 0.54)	0.93
gRM	−0.20 (−0.60, 0.20)	0.32
CYP2C9 phenotype (Ref = gNM)		
gIM	0.16 (−0.09, 0.41)	0.21
gPM	0.53 (−0.67, 1.73)	0.39
CYP3A4 phenotype (Ref = gNM)		
gIM	−0.08 (−0.44, 0.29)	0.67
CYP2C:TG haplotype present (Ref = absent)	−0.03 (−0.29, 0.22)	0.80
Female sex (Ref = male)	−0.02 (−0.25, 0.20)	0.83
Age (per year)	0.01 (0.00, 0.01)	0.30
Current smoker (Ref = non-/ex-smoker)	0.02 (−0.27, 0.31)	0.90
Number of drugs	0.03 (−0.01, 0.06)	0.21

Footnotes: Values are β coefficients with 95% confidence intervals calculated using HC3 heteroscedasticity-robust standard errors. Reference categories were gNM for genotype-predicted metabolizer phenotypes, absence of the *CYP2C:TG* haplotype, male sex, and non-smoking/former smoking. Age and number of drugs were entered as continuous variables.

Figure S1. Sertraline concentration-to-dose (C/D) ratios across combined *CYP2C19* and *CYP2B6* genotype-predicted metabolizer groups

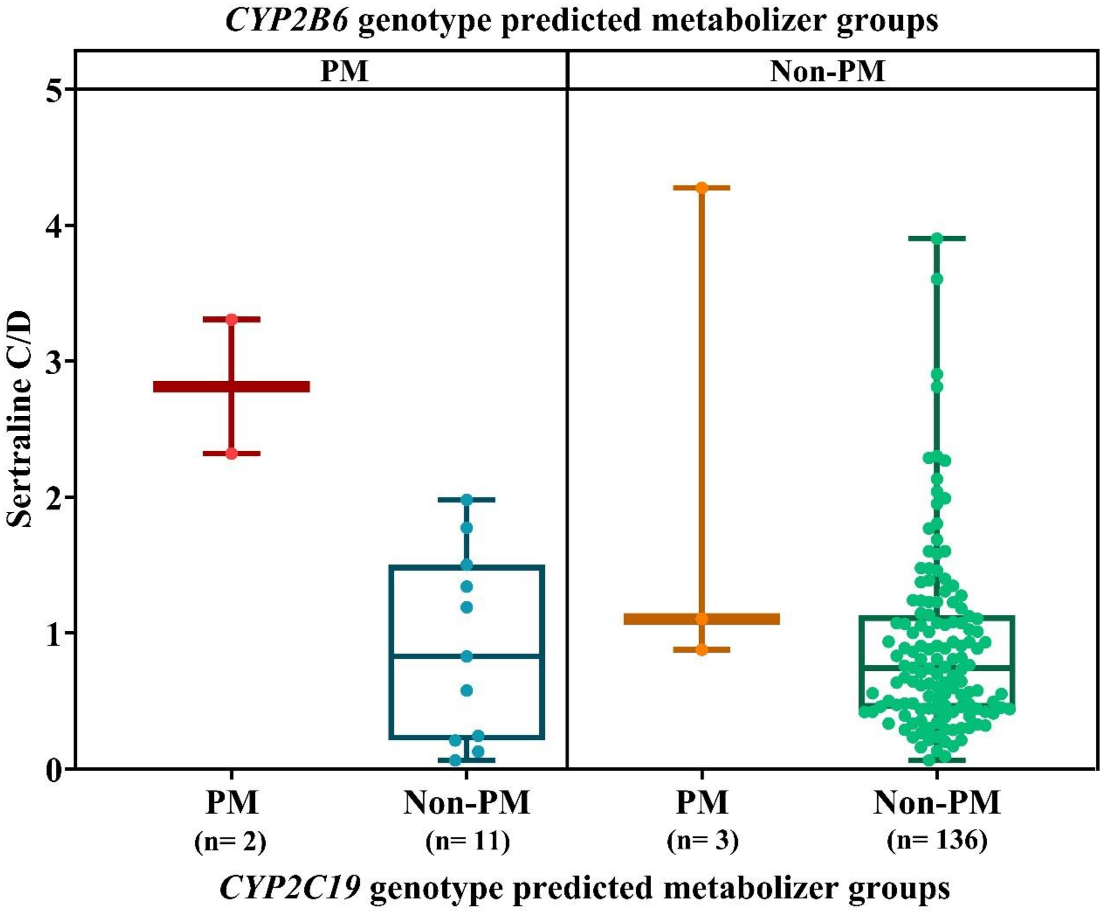

Footnotes: Boxes represent the interquartile range (25th –75th percentiles), horizontal lines within boxes indicate medians, and whiskers extend from the minimum to the maximum observed values. Dots represent individual patient observations. *CYP2C19* PM and *CYP2B6* PM groups were defined according to CPIC genotype-predicted metabolizer classifications. Non-PM includes genotype-predicted gIM, gNM, gRM, and gUM, as applicable. Subgroup sample sizes are provided below the corresponding x-axis labels. Owing to the very small number of participants with combined *CYP2C19* and *CYP2B6* PM status (n = 2), the analysis was descriptive, and no formal statistical comparisons were performed.

Figure S2. LogMR_{DN} across **A)** *CYP2C19*, **B)** *CYP2B6*, **C)** *CYP2D6,* **D)** *CYP2C9*, and **E)** *CYP3A4* genotype-predicted metabolizer groups

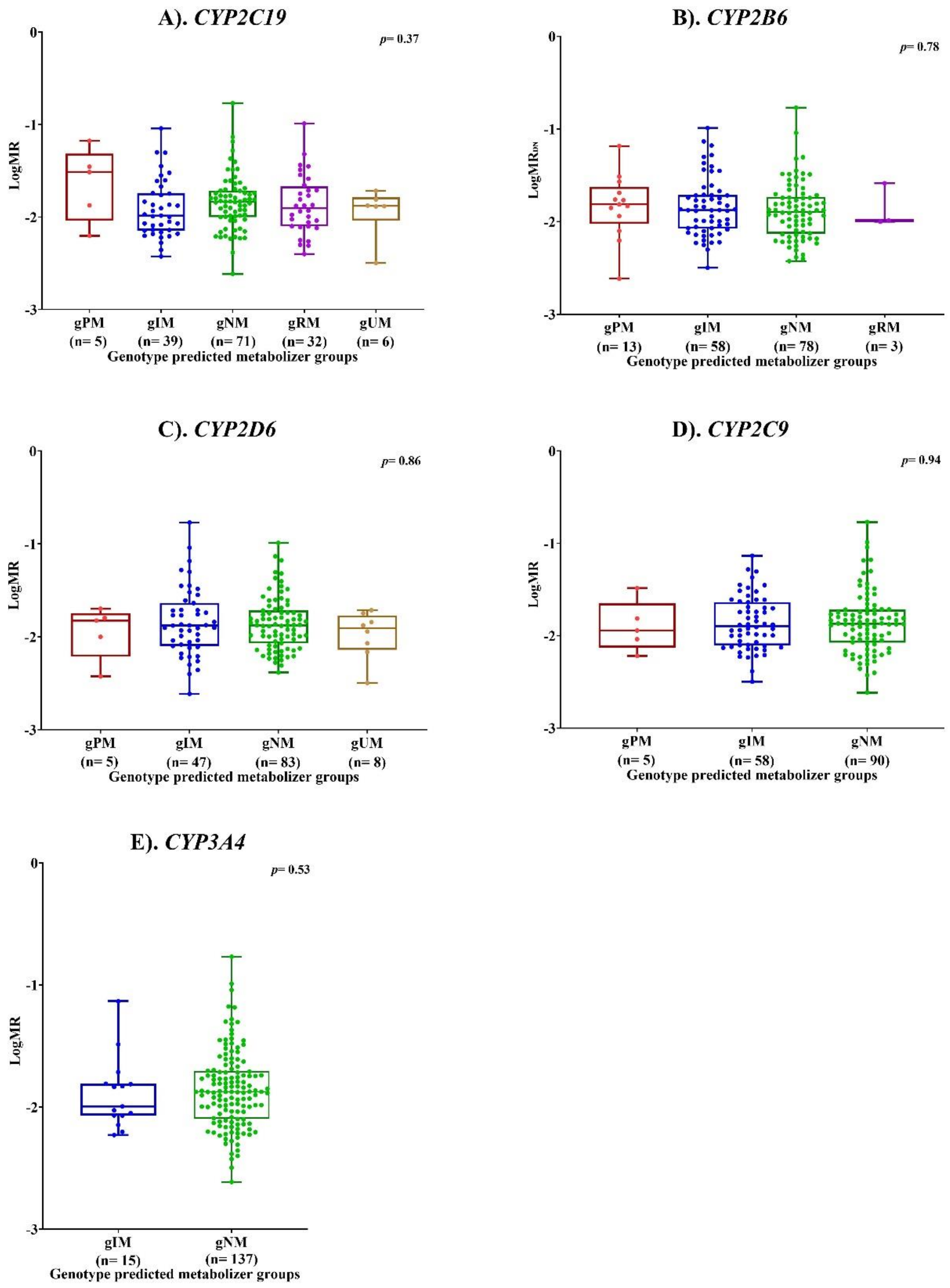

Footnotes: Genotype-predicted metabolizer status was indeterminate for 10 patients for *CYP2D6* and for 1 patient for *CYP2B6* and *CYP3A4.* Boxes represent the interquartile range (25th–75th percentiles), horizontal lines within boxes indicate medians, and whiskers extend from the minimum to the maximum observed values. Dots represent individual patient observations. Panels A-D were analyzed using the Kruskal–Wallis test, followed by Dunn's multiple-comparisons test, and panel E was analyzed using the Mann- Whitney test. Overall *p*-values are shown in the upper-right corner of each panel, while brackets indicate significant pairwise comparisons with *p*-values. Subgroup sample sizes are provided below the corresponding x-axis labels.

Figure S3. **(A)** Sertraline concentration-to-dose (C/D) ratio and **(B)** LogMR_{DN} are presented across *CYP2C19* genotype-predicted metabolizer groups stratified by *CYP2C:TG* haplotype status

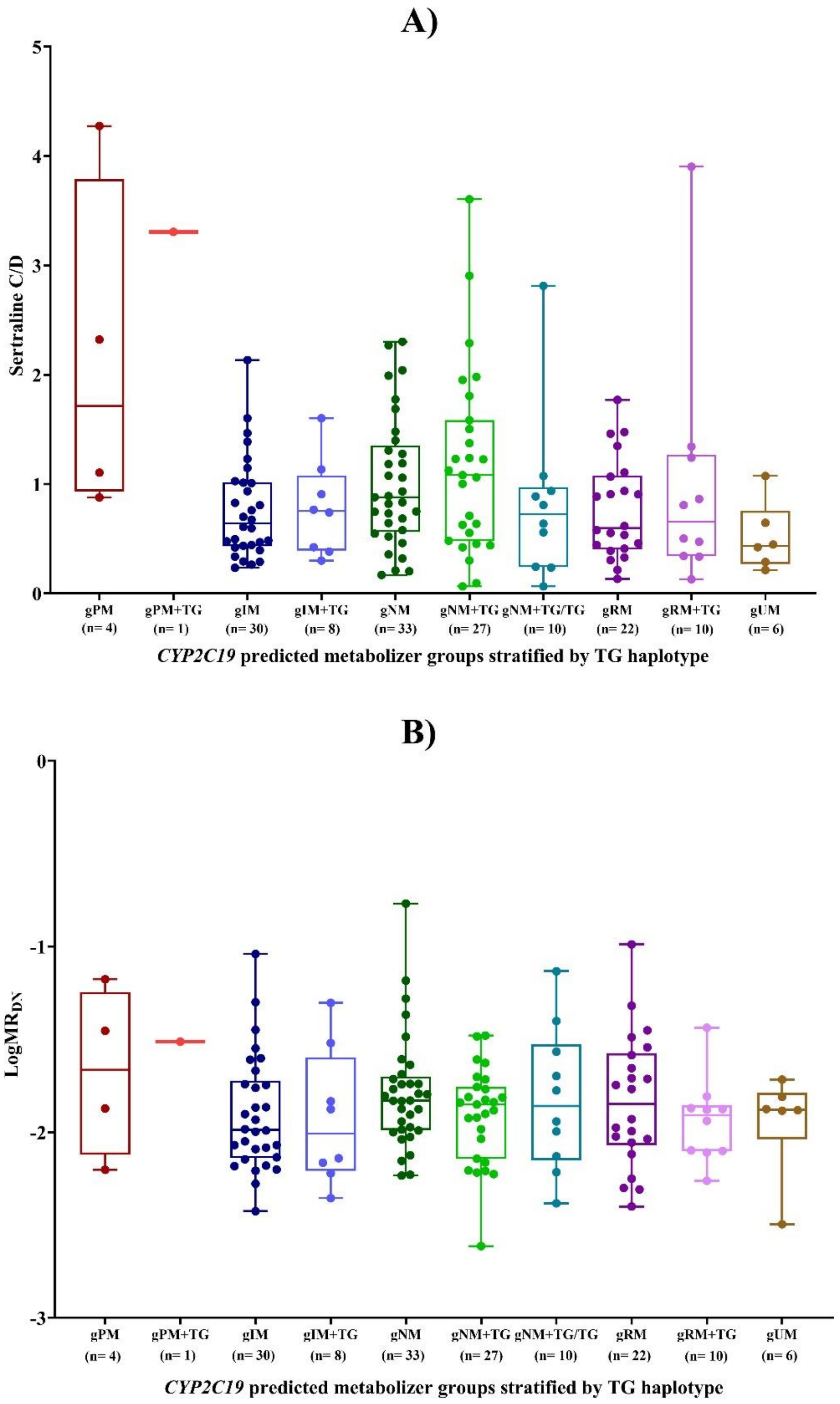

Footnotes: Boxes represent the interquartile range (25th–75th percentiles), horizontal lines within boxes indicate medians, and whiskers extend from the minimum to the maximum observed values. Dots represent individual patient observations. Subgroup sample sizes are provided below the corresponding x-axis labels.

Figure S4. Residuals *vs.* fitted values for the sertraline concentration model

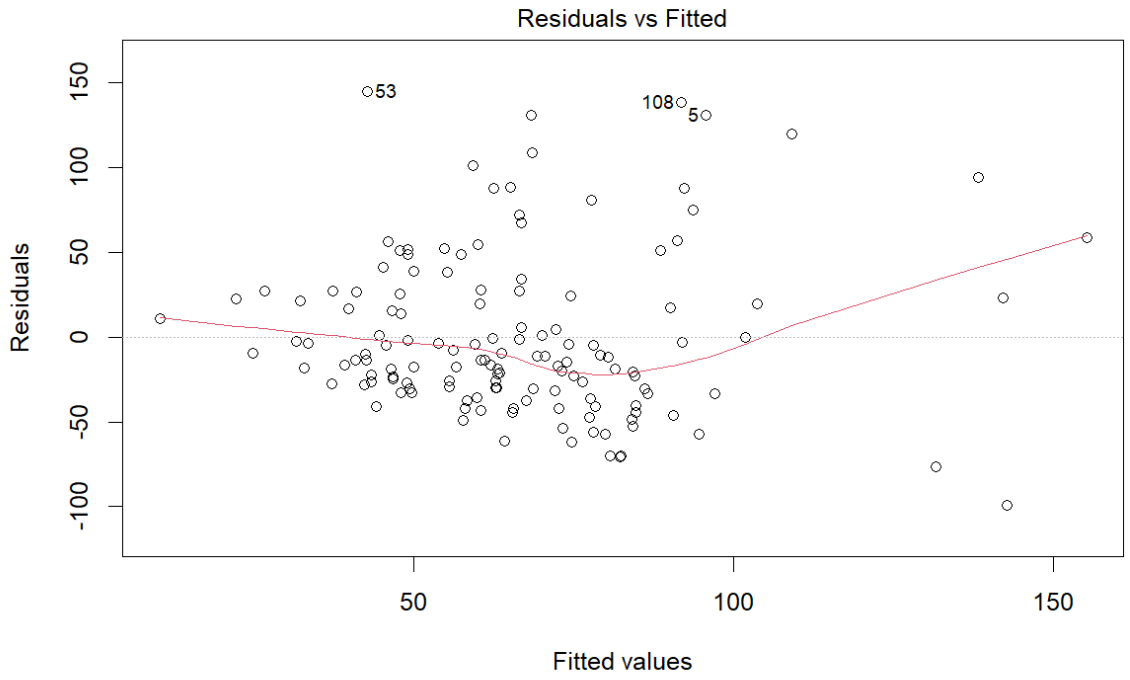

Figure S5. Normal Q-Q plot of standardized residuals for the sertraline concentration model

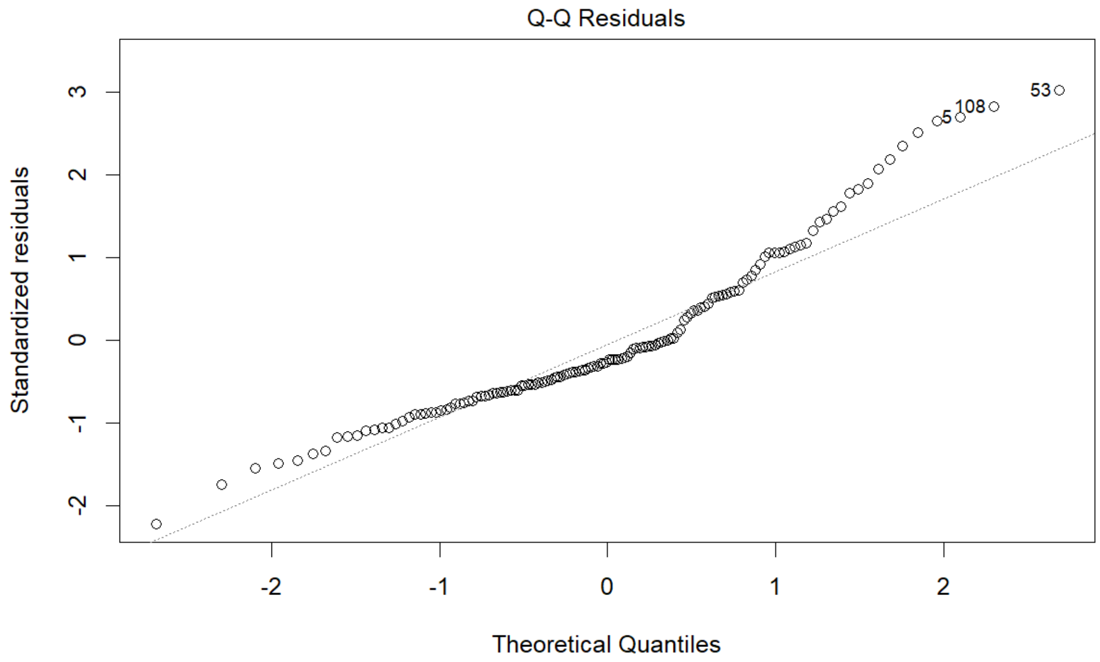

Figure S6. Residuals *vs.* fitted values for the sertraline concentration-to-dose ratio (C/D) model

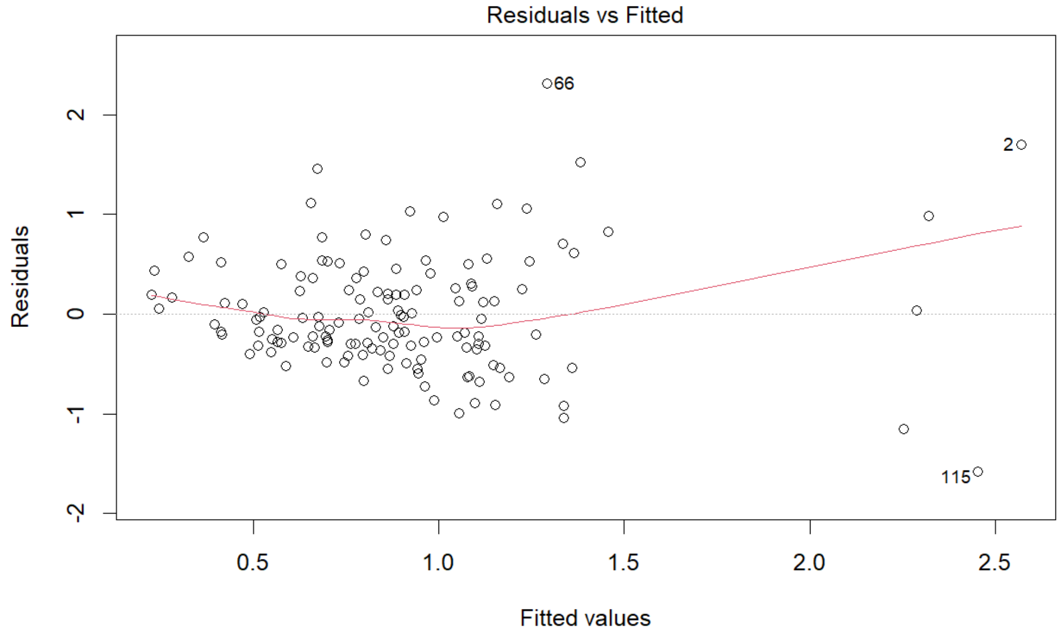

Figure S7. Normal Q-Q plot of standardized residuals for the sertraline concentration-to-dose ratio (C/D) model

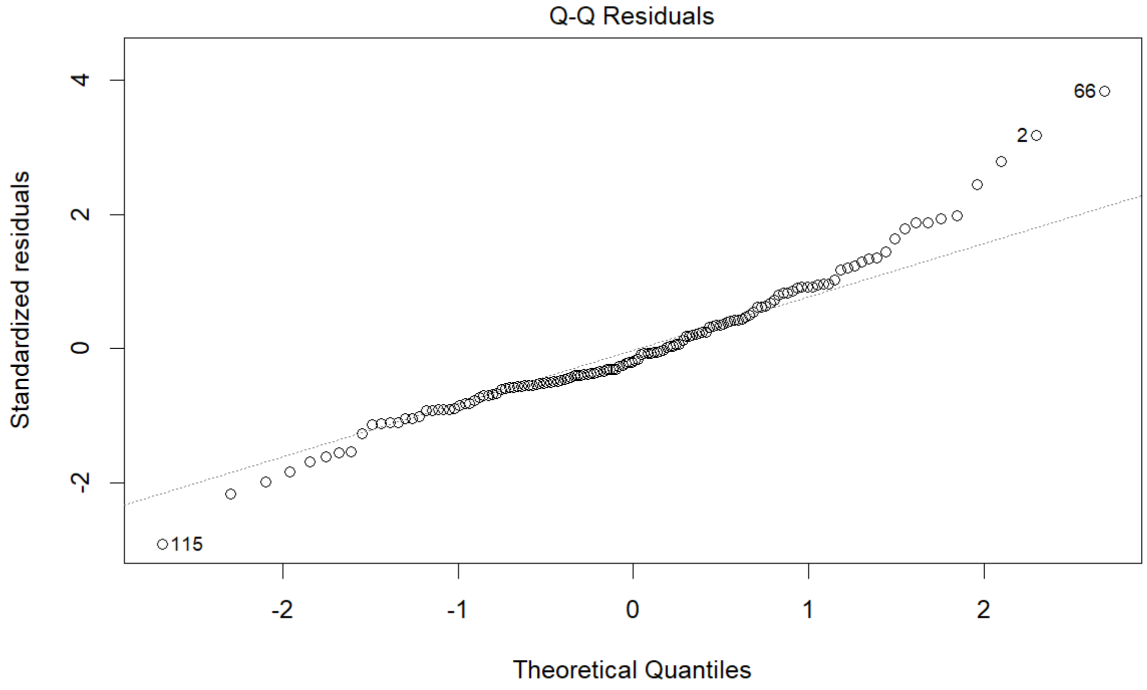

Figure S8. Residuals *vs.* fitted values for the metabolic ratio (MR) model

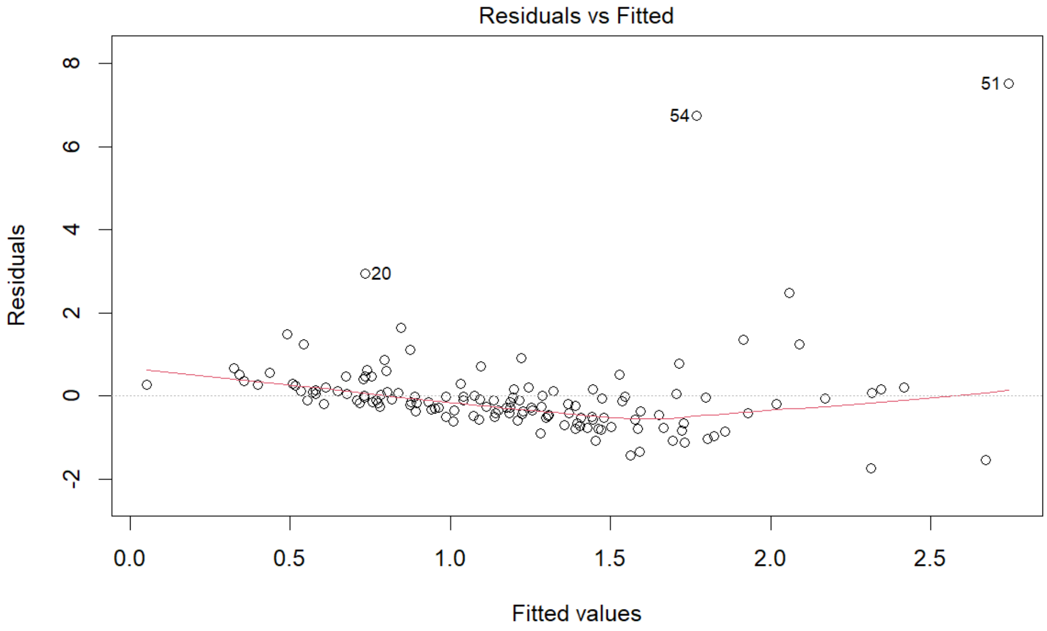

Figure S9. Normal Q-Q plot of standardized residuals for the metabolic ratio (MR) model

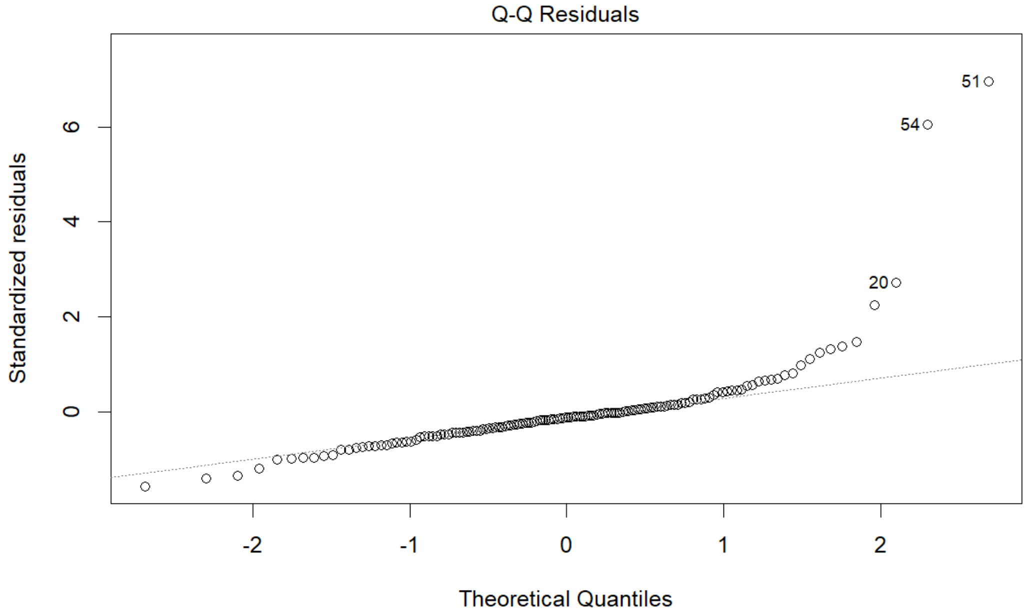

Figure S10. Residuals *vs.* fitted values for the $LogMR_{DN}$ model

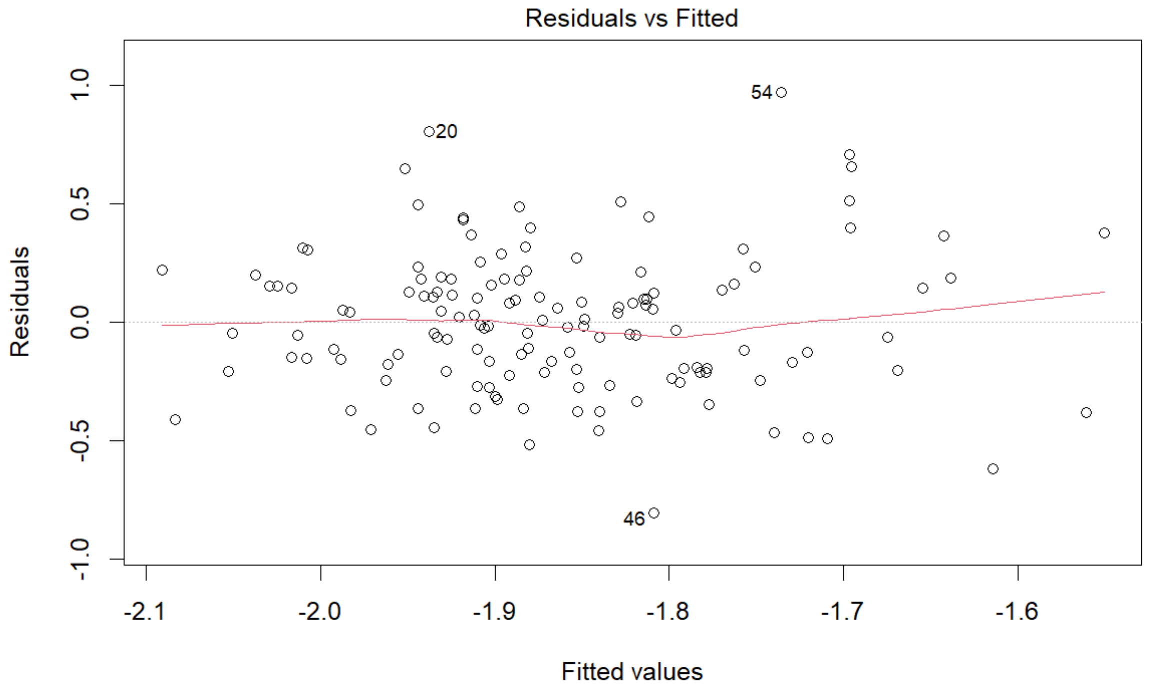

Figure S11. Normal Q-Q plot of standardized residuals for the $LogMR_{DN}$ model

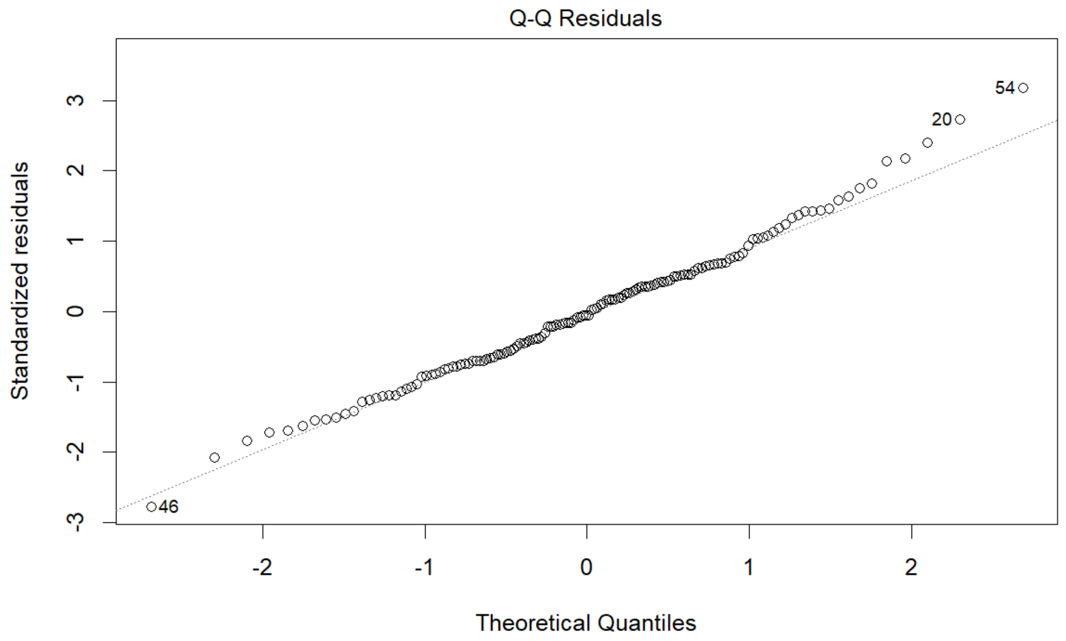

Figure S12. Residuals *vs.* fitted values for the LogMR model

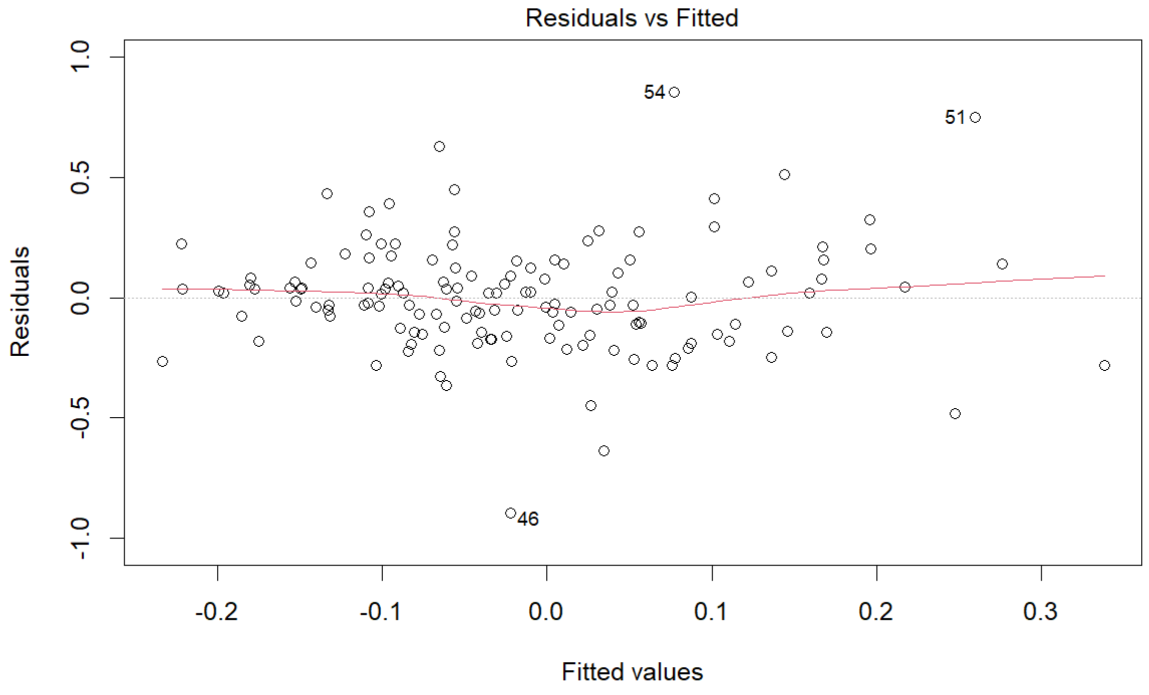

Figure S13. Normal Q-Q plot of standardized residuals for the LogMR model

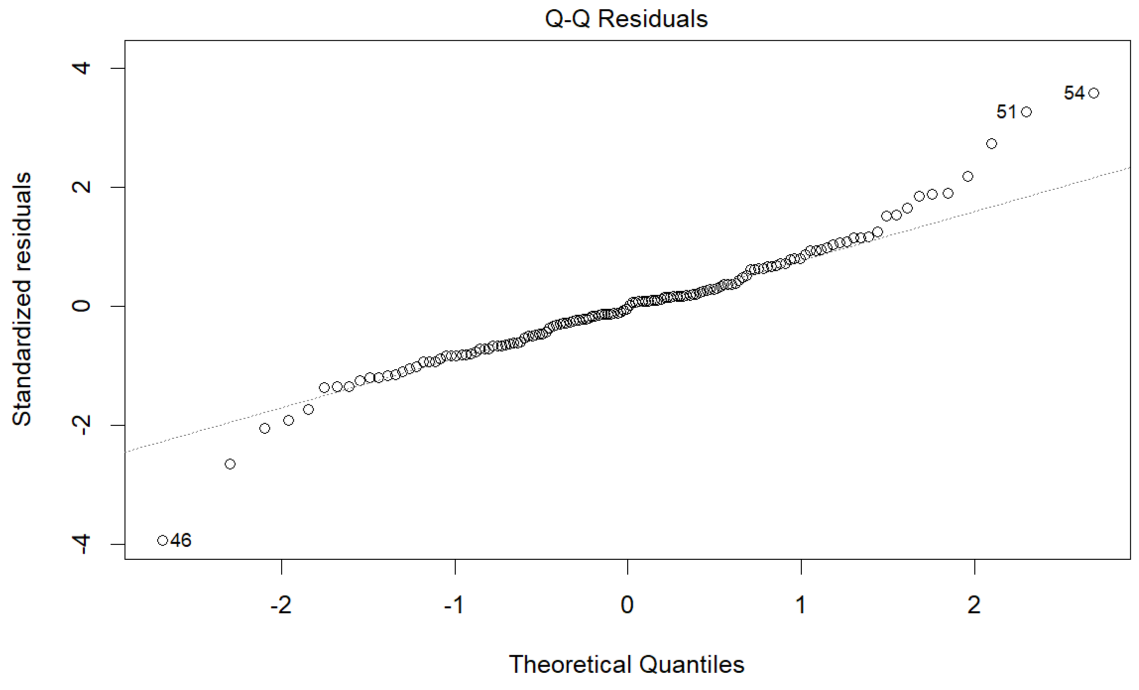

Supplement: Supplementary file 1 [file pharmaceutics-18-01133-s001.zip › pharmaceutics-4502580-supplementary.pdf]
